# Updated guidance on the management of cancer treatment-induced bone loss (CTIBL) in pre- and postmenopausal women with early-stage breast cancer

**DOI:** 10.1016/j.jbo.2021.100355

**Published:** 2021-03-18

**Authors:** Komal Waqas, Joana Lima Ferreira, Elena Tsourdi, Jean-Jacques Body, Peyman Hadji, M.C. Zillikens

**Affiliations:** a Bone Centre, Department of Internal Medicine, Erasmus University Medical Centre, Rotterdam, the Netherlands; b Department of Endocrinology, Hospital Pedro Hispano, Matosinhos Local Health Unit, Matosinhos, Portugal; c Department of Medicine III and 4. Center for Healthy Aging, Technische Universität Dresden Medical Center, Dresden, Germany; d Department of Medicine, CHU Brugmann, Université Libre de Bruxelles (ULB), Brussels, Belgium; e Frankfurt Center of Bone Health, Goethestrasse 23, Frankfurt, Germany and Philipps-University of Marburg, Germany

**Keywords:** IOF, International Osteoporosis Foundation, CABS, Cancer and Bone Society, ECTS, European Calcified Tissue Society, IEG, International Expert Group for AIBL, ESCEO: European Society for Clinical and Economics Aspects of Osteoporosis, Osteoarthritis and Musculoskeletal Diseases, IMS, International Menopause Society, SIOG, International Society for Geriatric Oncology, Aromatase inhibitors, Early-stage breast cancer, Fracture, Disease free survival, Bisphosphonates, Denosumab, Bone loss

## Abstract

**Introduction:**

Adjuvant endocrine therapy induces bone loss and increases fracture risk in women with hormone-receptor positive, early-stage breast cancer (EBC). We aimed to update a previous position statement on the management of aromatase inhibitors (AIs) induced bone loss and now included premenopausal women.

**Methods:**

We conducted a systematic literature search of the medical databases from January 2017 to May 2020 and assessed 144 new studies.

**Results:**

Extended use of AIs beyond 5 years leads to persistent bone loss in breast cancer extended adjuvant trials and *meta*-analyses. In addition to bone mineral density (BMD), vertebral fracture assessment (VFA) and trabecular bone score (TBS) were shown to independently predict fracture risk in real life prospective studies. FRAX® tool does not seem to be reliable for assessing fracture risk in CTIBL. In premenopausal women, there is strong evidence that intravenous zoledronate prevents bone loss but weak conflicting evidence on reducing disease recurrence from independent randomised controlled trials (RCTs). In postmenopausal women, the strongest evidence for fracture prevention is for denosumab based on a well-powered RCT while there is strong evidence for bisphosphonates (BPs) to prevent and reduce CTIBL but no convincing data on fractures. Adjuvant denosumab has failed to show anticancer benefits in a large, well-designed RCT.

**Discussion and conclusions:**

Extended use of AIs and persistent bone loss from recent data reinforce the need to evaluate fracture risk in EBC women initiated on AIs. Fracture risk should be assessed with clinical risk factors and BMD along with VFA, but FRAX is not adapted to CTIBL. Anti-resorptive therapy should be considered in those with a BMD T-score < −2.0 SD or with ≥ 2 clinical risk factors including a BMD T-score < −1.0 SD. In premenopausal women, intravenous zoledronate is the only drug reported to prevent bone loss and may have additional anticancer benefits. In postmenopausal women, either denosumab or BPs can be prescribed for fracture prevention with pertinent attention to the rebound phenomenon after stopping denosumab. Adjuvant BPs, in contrast to denosumab, have shown high level evidence for reducing breast cancer recurrence in high-risk post-MP women which should be taken into account when choosing between these two.

## Highlights

• Aromatase inhibitors induce bone loss and increase fracture risk in early-stage breast cancer women.
• BMD T-score < -2.0 SD or ≥ 2 clinical risk factors are an indication to start anti-resorptives.
• In pre-MP women, intravenous zoledronate is the only drug reported to prevent bone loss in EBC.
• In post-MP women, denosumab is a more efficient drug when fracture prevention is a concern and bisphosphonates should be preferred in case of high-risk of breast cancer recurrence.
• Sequential treatment with bisphosphonates after denosumab might mitigate rebound in bone turnover.

## 1 Introduction

Every year, 1.7 million women are diagnosed with breast cancer and 5-year overall survival (OS) is estimated to be 80% or higher in high-income countries, making it one of the most curable cancers in the world [1]. This increase in OS is related to improvements in early diagnosis due to the introduction of mammography screening along with improved treatment regimens for early breast cancer (EBC) [2]. As expected, breast cancer therapy does not lack side-effects among which increased fracture risk plays a significant role. Bone loss with breast cancer treatment occurs through several treatment modalities such as endocrine therapy [3], [4], [5], the topic of this review, but also through radiation, chemotherapy and its concomitant medications such as high-dose glucocorticoids [6]. Therefore, given the increasing number of breast cancer survivors, a practical approach is needed to prevent deterioration of bone quantity and quality, both leading to fragility fractures.

Adjuvant endocrine therapy (AET) with tamoxifen (TAM), gonadotropin-releasing hormone (GnRH) agonists and aromatase inhibitors (AIs) has been the mainstay of treatment in hormone receptor positive (HR + ) breast cancer [7], [8]. These therapies work by eliminating the effect of estradiol on breast tissue directly or indirectly but as a side-effect also on bone, thus leading to bone loss. With regard to bone tissue, TAM has been associated with opposing effects depending on the menopausal status. In premenopausal (pre-MP) women, TAM decreases bone mineral density (BMD) possibly by the competitive binding of a weak estrogen agonist (tamoxifen) against the stronger agonist (estradiol) on the estrogen receptors in bone. In post-menopausal (post-MP) women, tamoxifen has a bone protective or a neutral effect by acting as an estrogen agonist in bone when the levels of stronger agonist (estradiol) are extremely low [9], [10].

In 2017 a position paper of seven international bone and cancer societies was published on AI-induced bone loss (AIBL) [11]. In this current review, we analysed recently published data after 2017 on the use of AET and bone health in pre- and post-MP women with non-metastatic, hormone receptor positive (HR + ) breast cancer and included novel fracture risk assessment tools such as trabecular bone score (TBS) and vertebral fracture assessment (VFA) based on a systematic literature search strategy. The position statement [11] has provided an algorithm for management of AIBL based on clinical risk factors and BMD in HR+, EBC women. Based on the novel data from a systematic search, we aim to assess in this current review whether an update of the clinical management strategy as outlined in 2017 is necessary, as well as to evaluate how to address bone loss and fracture risk in pre-MP women with BC.

## 2 Search strategy

We performed a systematic literature search of the medical databases, including Embase, Medline and Cochrane Central from January 2017 to May 2020. The search criteria used the following key words: “Adjuvant endocrine therapy for breast cancer” OR “GnRH agonists” OR “Tamoxifen” OR “Aromatase inhibitor” OR “anastrozole” OR “exemestane” OR “letrozole” **AND** “bone health” OR “fracture” OR “bone mineral density” OR “bisphosphonates” OR “denosumab”**.**

**Fig. 1:**
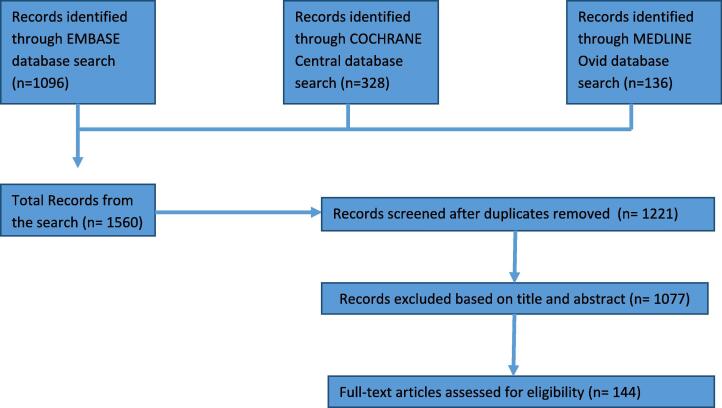
**Flowchart showing criteria for inclusion and exclusion of studies**.

Among those 1221 identified citations, we considered as high-quality papers those reporting on well-powered randomized controlled trials (RCTs) and/or *meta*-analyses, but we also collected evidence from observational case-control studies and reviews which could be regarded as low level evidence. During preparation of this manuscript, one relevant and recently published article was identified and also included. At the end, a total of 144 papers were assessed to be included in this review; see Fig. 1 and supplementary table 1 for the funnelling of the search criteria and number of the articles assessed per topic Table 1, Table 2

**Table 1:** Major studies and updates from January 2017 to May 2020 regarding anticancer benefits of antiresorptive agents in women with EBC.

	**Study**	**Population at study entry, N**	**Intervention, n**	**FU, M**	**Dose, route of administration**	**Disease-free survival**	**Overall survival**
**Bisphosphonates**	Perrone *et al*, 2019, HOBOE trial [20]	Pre-MP HR + BC Adj triptorelin Median age: 45y N = 1065	Tamoxifen = 354 vs. Letrozole = 356 vs. Zoledronate + letrozole = 355 Treatment duration 5y	64	ZOL 4 mg, Q6M. IV	At 5y: 85.4% vs. 93.2% vs. 93.3% (p = 0.008) DFS events (n = 134): 16.4% vs. 12.4% vs. 9.0% - Zoledronate + Letrozole vs. Tamoxifen: HR 0.52, 95% CI = 0.34–0.80, p = 0.003 - Zoledronate + Letrozole vs. Letrozole: HR 0.70, 95% CI = 0.44–1.12, p = 0.22	Deaths (n = 36): 4.8% vs. 3.1% vs. 2.3%, p = 0.14
	Livi *et al*, 2019, BONADIUV trial [99]	Post-MP Osteopenic HR + BC, Adj AI Median age: 60y N = 171	Ibandronate = 89 vs. Placebo = 82 Treatment duration 2y	63	150 mg, Q4W. Oral	At 5y: no difference (p = 0.42)	OS 93% vs. 97.5%, p = 0.19
	Gralow *et al*, 2019 SWOG S0307 trial [74]	Pre- and post-MP Stage I-III BC Median age: 52.7y N = 6018	ZOL = 2231 vs. Ibandronate = 2235 vs. Clodronate = 1552 Treatment duration 3y	60	4 mg, Q1M x6; Q3M × 10. IV 50 mg, Q1D. Oral 1600 mg, Q1D. Oral	At 5y: 88.3% vs. 87.4% vs. 87.6%, p = 0.49	At 5y: 92.6% vs. 92.9% vs. 92.4%, p = 0.50
	Coleman *et al*, 2019, AZURE (BIG 01/04) trial [100]	Stage II-III BC Ad AI (55.5%) Median age: NA N = 3359	ZOL = 1681 vs. Standard = 1678 Treatment duration 5y	117	4 mg, Q4W x6; 4 mg, Q3M x8; 4 mg, Q6M x5. IV	At 117 M: DFS events 555 vs.575; (HR 0.94, 95% CI = 0.84–1.06)	OS: 69% vs. 64.6% (HR 0.92, 95% CI = 0.81–1.05) In > 5y post-MP: HR 0.84, 95% CI = 0.67–1.04
**Denosumab**	Gnant *et al*, 2019, ABCSG-18 [77]	Post-MP HR + BC Adj AI Median age: 64y N = 3420	Dmab = 1711 vs. Placebo = 1709 Treatment duration 5y	73	60 mg, Q6M. Subcutaneous	At 5y: 89·2% vs. 87.3% At 8y: 80.6% vs. 77.5% HR 0.82, 95% CI = 0.69–0.98, p = 0.026	–
	Coleman *et al*, 2018, D-CARE study [79]	Pre- and post-MP 77% HR + II-III BC Median age: 51y N = 4509	Dmab = 2256 vs. Placebo = 2253 Treatment duration 5y	67	120 mg, Q4W x6; 120 mg, Q3M x54. Subcutaneous	DFS (n = 875): 1.04, 95% CI = 0.91–1.19, p = 0.57 BM (n = 597): 0.97, 95% CI = 0.82–1.14, p = 0.70	OS (n = 412): HR 1.03, 95% CI = 0.85–1.25

Adj, adjuvant; AI, aromatase inhibitor; BC, breast cancer; BMD, bone mineral density; BMs, bone metastasis; D, day; DFS, disease-free survival; Dmab, denosumab; FU, follow-up; HR, Hazard ratio; HR+, hormone-receptor positive tumours; IV, intravenous; M, month(s); NA, not available; OS, overall survival; Q, every; W, week; y, year; ZOL, zoledronic acid.

**Table 2:** Major studies and updates from January 2017 to May 2020 regarding bone loss and fracture prevention of antiresorptive agents in women with EBC.

	**Study**	**Population at study entry, N**	**Intervention, n**	**FU, M**	**Dose, route of administration**	**Mean BMD/T-score change from baseline, %**		**Fracture data**
						**LS**	**TH or FN**	
**Bisphosphonates**	Wilson *et al*, 2018, AZURE trial [50]	Stage II-III BC Adj AI (55.5%) Median age: NA N = 3359	ZOL = 1681 vs. Controls = 1678	84	4 mg, Q4W x6; 4 mg, Q3M x8; 4 mg, Q6M x5. IV	–	–	5y rate: 3.8% vs. 5.9%; Time to first fracture: HR 0.69, 95% CI = 0.53–0.90, p = 0.005
	Santa-Maria *et al*, 2018, ZAP trial [101]	Post-MP Stage 0-III BC Adj AI Median age: 59y N = 262	ZOL + L (ZAP trial) = 59 vs. L (ELPh trial) = 203	12	4 mg, Q6M. IV	T-score: +0.23, 95% CI = 0.13–0.33, p < 0.001 (12 M)	T-score: +0.12, 95% CI = 0–0.23, p = 0.046 (12 M)	–
	Sestak *et al*, 2019, IBIS-II Bone substudy [102]	Post-MP Osteopenic At high risk of BC Median age: NA N = 127	Risedronate = 68 vs. Placebo = 59	60	35 mg, Q1W. Oral	T-score: −0,4% vs. −4.2% p < 0.0001	T-score: −2.5% vs. −3.8%, p = 0.2	No difference in rate (20 vs. 18; RR = 0.91 (0.46 vs. 1.81)
	Livi *et al*, 2019, BONADIUV trial [99]	Post-MP Osteopenic HR + BC Adj AI Median age: 60y N = 171	Ibandronate = 89 vs. Placebo = 82	63	150 mg, Q4W. Oral	T-score: +0.35 vs. −0.24, p < 0.0001 (24 M)	T-score: +0.28 vs. −0.09, p = 0.0002 (24 M)	–
	Monda *et al*, 2017 [103]	Post-MP Osteopenic HR + EBC Adj AI Mean age: 56y N = 84	Risedronate = 42 vs. No treatment = 42	24	35 mg, Q1W. Oral	T-score: +6.86% vs. −4.8%, p < 0.0001	T-score: +2.8% vs. −3.5% p < 0.0001	Fractures: 0 vs. 3 (short FU and relatively young age)
**Denosumab**	Nakatsukasa *et al*, 2019 [65], [67]	Post-MP Osteoporotic HR + I-IIIA BC Adj AI Mean age: 65y N = 103	Dmab = 93 (nonrandomized)	24	60 mg, Q6M. Subcutaneous	BMD: +7.0, 95% CI = 5.9–8.0 (24 M)	BMD: +3.4% to + 3.6% (24 M)	Any symptomatic clinical fractures (24 M)

Adj, adjuvant; AI, aromatase inhibitor; BC, breast cancer; BMD, bone mineral density; Dmab, denosumab; FN, femoral neck; FU, follow-up; HR, Hazard ratio; HR+, hormone-receptor positive tumours; IV, intravenous; LS, lumbar spine; M, month(s); NA, no available; Q, every; RR, relative risk; TH, total hip; W, week; y, years; ZOL, zoledronic acid.

## 3 Literature evidence

### 3.1 Adjuvant endocrine therapy and evidence related to bone loss and fractures

#### 3.1.1 Premenopausal women

Overall, several head-to-head RCTs comparing TAM with AIs/OFS in pre-MP women with EBC reported an increased bone loss with both classes of drugs with an annual BMD loss up to 11% with AIs/OFS, due to a profound suppression of oestrogen production. Tamoxifen causes less, but significant decrease in BMD (up to 2% per year) [12], [13], [14], [15]. There is also a deterioration of bone microarchitecture, as confirmed by TBS (2% decrease after 24 months of AET) [16], [17]. Fractures in pre-MP women are either reported in RCTs as adverse events or observed in case-control studies. In the past, studies such as the ABCSG-12 trial found no difference in fracture rates between pre-MP women treated with chemical castration and AIs or TAM at 62 months of follow-up [18]. The updated analysis of two crucial RCTs, the Tamoxifen and Exemestane Trial (TEXT) and the Suppression of Ovarian Function Trial (SOFT) from 2019 reported approximately a two-fold increased risk of osteoporosis (14.8% vs. 7.2%) and an increased number of fractures during treatment (7.7% vs. 6.0%) with an AI/OFS compared to a TAM/OFS combination [19]. The HOBOE trial (n = 1067) in pre-MP women with median age 45yrs at randomisation reported no cases of fractures at 60 months of follow-up, collected as adverse events [20]. In a prospective case-control study, pre-MP women with EBC were compared to healthy controls (n = 1761) with regard to their cumulative fracture incidence (based on ICD codes) up to 5 years of follow-up. The authors observed higher hazards of fracture incidence (hazard ratio HR 2.67, 95% CI = 1.58–4.53) in women on tamoxifen (n = 1120) vs. healthy controls and no difference in fracture incidence (HR = 1.63, 0.80–1.33) in women without tamoxifen (n = 533) vs. healthy controls [10]. Another recent case-control study reported a 75% higher fracture incidence (HR 1.75, 1.25–2.48) in pre-MP women on tamoxifen (n = 1817) vs. non-breast cancer controls (n = 1817) [21]. However, these findings need to be confirmed in dedicated RCTs in pre-MP women with an optimally long follow-up period.

#### 3.1.2 Postmenopausal women

In post-MP women, AIs have been associated with increased bone turnover and bone loss [22], [23], [24], [25], and increased fracture risk [26], [27], [28], [29], [30] in comparison to TAM. A *meta*-analysis from 2011, including 7 RCTs (n = 30,023), reported a 47% higher risk of fractures (odds ratio, OR 1.47, 95% CI = 1.34–1.61, p < 0.001) with a longer duration of AIs use [31]. A similar increase in fracture risk of 35% with AIs was reported in a recent *meta*-analysis (n = 20,403) [32] and 40% (HR 1.40, 95% CI = 1.05–1.87) in a large-scale real-world, cohort study (n = 36,472), both compared to TAM [33]. Because of ethical reasons, no placebo controlled RCTs with AIs vs. placebo have been performed but only head-to-head trials with TAM in post-MP women.

**Fig. 2:**
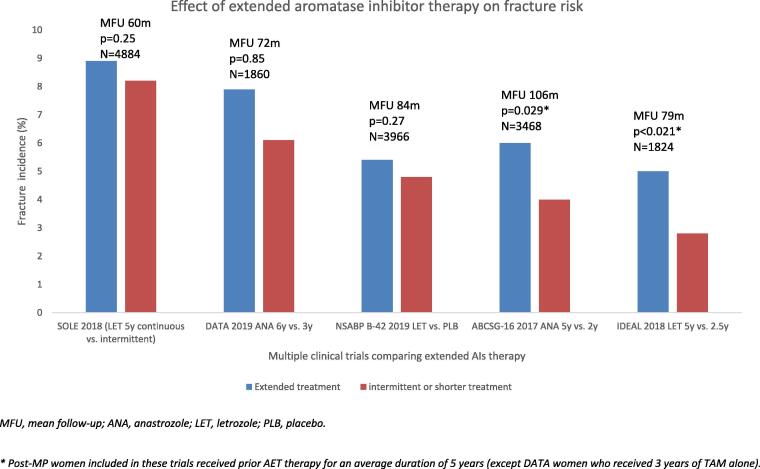
**Comparison of the major trials on extended AI therapy published between 2017 and 2019 and fracture risk** (after first 5 years of treatment with adjuvant endocrine therapy). MFU, mean follow-up; ANA, anastrozole; LET, letrozole; PLB, placebo. * Post-MP women included in these trials received prior AET therapy for an average duration of 5 years (except DATA women who received 3 years of TAM alone).

Breast cancer trials (NSABP B-42, DATA, IDEAL, MA.17R and ABCSG-16) comparing extended duration of AIs to placebo or no treatment further confirmed increased fracture risk as a secondary outcome due to AIs use (see Fig. 2) [34], [35], [36], [37]. This has further been confirmed in a *meta*-analysis from 2018 including seven RCTs (n = 16,349) which reported higher odds of fractures (OR = 1.34, 95% CI = 1.16–1.55) with extended AIs therapy compared to placebo or no treatment [38].

### 3.2 Intervention in adjuvant endocrine therapy induced bone loss in EBC women

#### 3.2.1 Non-pharmacological

Evidence regarding non-pharmacological measures such as calcium and vitamin D use in EBC to improve bone health is lacking. Likewise, the evidence on the role of exercise on improving bone health specifically in breast cancer survivors is relatively scarce. A systematic review and *meta*-analysis from 2017 including 1199 patients with EBC from 10 RCTs evaluated the effect of 12-month exercise programs on BMD. It was found that resistance training or impact exercises did not improve mean changes in BMD at the lumbar spine (LS), femoral neck (FN) or total hip (TH) at the end of the 12-month period in post-MP women [39]. In one relatively large RCT (n = 498) including pre-MP women (n = 229) with breast cancer, supervised weight-bearing jumping exercises and circuit training for 12 months prevented BMD loss at FN (−0.2% vs. − 1.4%), but not at LS (−1.9% vs. − 2.2%) [40].

Since 2017, three small scale (n = 41 to 121) RCTs have been published comparing the effect of exercise vs. usual care on BMD, bone turnover markers and/or body composition in breast cancer survivors [41], [42], [43]. These trials reported that resistance and aerobic training consistently led to a reduction in total body fat, a trend towards stimulation of bone formation, evaluated by osteocalcin or bone specific alkaline phosphatase, and possibly an improvement in lean mass with longer duration and intensity of these training programs. However, there were no changes reported in BMD at any site possibly due to short follow-up (12 weeks to 12 months). There is also increasing evidence that exercise leads to improvement in quality of life and breast cancer-related outcomes [44], [45]. This initial evidence needs further confirmation in RCTs with longer follow-up duration and including endpoints like fractures. However, extrapolating from these data, we should encourage exercise in women with EBC not only to improve bone health but also because of multiple other potential benefits.

#### 3.2.2 Pharmacological

Bisphosphonates (BPs) and Denosumab (Dmab) represent the major bone-targeted therapies to counteract bone loss. Anabolic drugs, such as PTH-analogues or romosozumab are not recommended in women with EBC due to lack of evidence. For PTH-analogues namely teriparatide or abaloparatide, there is a concern about the potential risk of osteosarcoma based on preclinical studies [46], [47] and the drug leaflet carries a warning for those who have an established metastasis or who have received radiation therapy.

### 3.3 Prevention and treatment of bone loss and fractures

#### 3.3.1 Premenopausal women

Data on adjuvant anti-resorptive therapy in preventing bone loss as a primary end-point in pre-MP women with EBC is limited. Intravenous zoledronic acid (iv ZOL, 4 mg, every (Q) 3–6 months) is the only BP which has been shown to prevent BMD loss in pre-MP women on AET. Strong evidence derived from the Austrian Breast and Colorectal cancer study group 12 trial (ABCSG-12), where concurrent ZOL with AET countered bone loss in pre-MP women (n = 404) compared with those without ZOL who showed an ongoing bone loss during AET (At 3 years, ZOL vs. no ZOL: +0.4% vs. −11.3% at lumbar spine and + 0.8% vs. −7.3% at trochanter) [12]. Other relatively small-scale RCTs with ZOL and either AIs/OFS, TAM/OFS or TAM alone strengthen the evidence for prevention of BMD loss in pre-MP women [48], [49].

The only evidence on fracture prevention in pre-MP women came from the AZURE trial in 2018, reporting data on fractures as a secondary end-point. Here the addition of ZOL to (neo)adjuvant chemotherapy and/or endocrine therapy in a group of pre-, *peri*-menopausal or women with unknown menopausal status (n = 1507) showed a fracture incidence of 2.9% in the ZOL arm and 4.2% fractures in the control arm at 5-years and increased time-to-first fracture (HR, 95% CI = 0.45–0.88) [50]. There are no clinical trials or studies on using adjuvant oral BPs or Dmab in pre-MP women on endocrine therapy. In summary, there is strong evidence that adjuvant ZOL in pre-MP women can prevent bone loss and suggestive evidence that ZOL may decrease fracture risk.

#### 3.3.2 Postmenopausal women

To date, fracture data as a dedicated primary endpoint in RCTs performed in post-MP women initiated on AIs and adjuvant anti-resorptive therapy are scarce. Most of the trials reported favourable BMD changes from baseline, instead of fractures, when comparing the efficacy of adjuvant intravenous BP vs. placebo/no treatment in AIBL [51], [52], [53], as also reported in the previous position statement in 2017 [54], [55], [56].

**Oral bisphosphonates** have been reported in various trials before 2017 to reduce bone loss in AIs users as compared to placebo [57], [58], [59], [60], [61]. A few small-scale trials after 2017 (n ranging from 81 to 171) using risedronate or ibandronate vs. placebo confirmed improvement in BMD (up to + 6.0% at LS at 24 months) in post-MP women while on AIs and added to current body of evidence. Fracture data for oral BPs has been reported in 2019 in a large-scale observational cohort study (n = 36,472) with a mean follow-up of 10 years in which the incidence rate of fracture was 30% lower in oral BP-treated patients within the AIs high-risk subgroup (with a diagnosis of osteoporosis) than in patients without BPs (HR 0.69, 95% CI = 0.48–0.98) [62].

Use of the **intravenous bisphosphonate** zoledronate (ZOL), provided strong evidence of cumulative BMD increase from baseline to around 6% at LS and 2.6% at total hip (TH) during a maximum follow-up of 60 months [63]. In a secondary end-point analysis in 2018, the AZURE trial (n = 3359) reported the 5-year fracture rate to be significantly reduced (3.9% vs. 5.8%) in women receiving adjuvant ZOL vs. controls during AET. A larger reduction in fracture rate occurred after disease recurrence (ZOL vs. controls: 2.8% vs. 9.8%) than before disease recurrence (3.8% vs. 3.3%)[50]. Although there is fracture reduction with ZOL in these data, it seems less pronounced than expected from BMD changes in previous studies. Although studying fractures as primary end-point in clinical trials remains ideal, data from *meta*-regression of trials [64] provide profound evidence that improvements in BMD can be a useful surrogate endpoint for fractures.

**Denosumab** Few studies have been published regarding the role of adjuvant Dmab (60 mg every 6 months) in bone health improvement in EBC post-MP women. Ellis and colleagues reported in 2008 a sustained increase in LS-BMD (7.6%) at 24 months in those receiving Dmab vs. placebo. Increase in LS-BMD (up to 7% at 24 months) with adjuvant Dmab has also been corroborated in recent prospective, non-randomized studies in Japanese osteoporotic and osteopenic post-MP women on AIs [65], [66], [67]. A well-powered, high-quality evidence to study fractures as a primary end-point was ascertained only using Dmab. In the ABCSG-18 trial, a 50% reduced incidence of clinical fractures was reported in post-MP women on adjuvant Dmab compared to placebo at 84 months [11.1%(8.1–14.1) in Dmab vs. 26.2%(15.6–36.8) in placebo group], irrespective of their baseline BMD or age [68].

### 3.4 Additional survival benefits

Preclinical studies suggested a role of accelerated bone remodelling in dissemination of tumour cells [69]. Extrapolation of this principle combined with recent studies including the EBCTCG *meta*-analysis [70] underline the clinical role of bisphosphonate therapy to prevent breast cancer recurrence and dissemination to bone and has been acknowledged in several international guidelines [71].

#### 3.4.1 Premenopausal women

Evidence on anticancer benefits of adjuvant ZOL in pre-MP women with EBC derives from RCTs (two dating before and one after 2017). In the ABCSG-12 from 2012, a 36% reduction in disease recurrence was reported with ZOL + endocrine therapy vs. endocrine therapy alone in all pre-MP women receiving OFS [18]. Conversely, in the AZURE trial where pre-MP women constituted 45% of the population with almost half receiving an AI, there was no benefit found in invasive disease free survival (iDFS) in ZOL vs. placebo (HR 1.03, 95% CI = 0.89–1.20). The HOBOE, a three-arm RCT, compared the effects of TAM/OFS vs. Letrozol/OFS (L) +/- ZOL on survival benefits in 1065 pre-MP women [20]. Comparing three arms simultaneously, there was no statistically significant difference in overall survival (OS) (TAM: 4.8% vs. L: 3.1% vs. L + ZOL: 2.3%) and DFS (TAM: 16.4% vs. L: 12.4% vs. L + ZOL: 9.0%) but a suggestive trend probably due to extremely low number of events. By combining the HOBOE and ABCSG-12 trials, there was a significant improvement in DFS (HR 0.75, 95% CI = 0.60–0.94) when ZOL plus endocrine therapy was compared with endocrine therapy alone in pre-MP women. These independent RCTs provide weak conflicting evidence on reducing disease recurrence in pre-MP women on AIs/OFS while suggesting a need for more dedicated RCTs.

#### 3.4.2 Postmenopausal women

##### 3.4.2.1 Oral and intravenous bisphosphonates

The most convincing evidence, pertaining to the use of oral (clodronate mainly, ibandronate partially) and intravenous BPs, for preventing breast cancer recurrence came from the EBCTCG *meta*-analysis in post-MP women (n = 11,767). Adjuvant BPs led to significant reductions in overall recurrence (RR 0.86, 95% CI = 0.78–0.94, p = 0.002), bone recurrence (RR 0.72, 95% CI = 0.60–0.86, p = 0.0002) and breast cancer mortality (RR 0.82, 0.73–0.93, p = 0.002) [72]. In a recent update of the AZURE trial (2019) designed to compare the effect of ZOL to standard therapy, ZOL improved invasive DFS (HR 0.78, 95% CI = 0.64–0.94) but not OS (0.84, 95% CI = 0.67–1.04) in a subgroup of women that were longer than 5 years post-MP. Very recently, a large scale retrospective cohort study on women aged ≥ 66 years (n = 37,724) revealed an improved OS (HR = 0.87, 95% CI = 0.82–0.93 and breast cancer specific survival (HR = 0.77, 95% CI = 0.64–0.92) in those receiving bisphosphonates (n = 6898) at osteoporosis doses during the first 2 years after cancer diagnosis [73] when compared to no treatment. These are in line with the previously published EBCTCG *meta*-analysis [72].

A direct head-to-head comparison of adjuvant ZOL, daily clodronate and daily ibandronate for 3 years reported no differences according to the type of BPs even after 5-years: DFS rate [88.3% for ZOL vs. 87.6% for clodronate vs. 87.4% for ibandronate] and OS rate [92.6% for ZOL vs. 92.4% for clodronate vs. 92.9% for ibandronate] [74]. Routinely used oral BPs (alendronate and risedronate) have been reported mainly in population-based case-control studies to improve breast cancer survival [75], [76] but there have been no dedicated RCTs.

##### 3.4.2.2 Denosumab

Regarding anticancer effects of **denosumab**, the ABCSG-18 trial compared DFS as a secondary endpoint between Dmab (60 mg, every 6 months (Q6M)) and placebo arms in 3420 post-MP EBC women on AIs. At a median follow-up of 96 months, Dmab was associated to higher DFS compared with placebo (80.6% vs. 77.5%, p = 0.025). Nevertheless, a closer look revealed that the majority of the DFS benefits in ABCSG-18 were due to a reduction in histologically verified second primary invasive non-breast carcinoma in the Dmab group, with little effect on contralateral or distant breast cancer recurrence [77].

Another recently published phase 3 RCT (D-CARE) with DFS as a primary end-point evaluated the addition of adjuvant Dmab (120 mg, Q1M for the first 6 months, Q3M thereafter) to standard (neo)adjuvant therapy in 4509 high-risk EBC women (77% HR+, 20% Her2 + ). At a median follow-up of 67 months, results showed no benefits of Dmab on DFS (HR 1.04, 95% CI = 0.91–1.19) and OS (HR 1.03, 95% CI = 0.85–1.25) compared with placebo [78], [79]. Also, a recent large scale (n = 37,724) retrospective cohort study observed neither an improvement in OS (HR = 1.05, 95% CI = 0.90–1.22) nor breast cancer specific survival (HR = 1.09, 95% CI = 0.66–1.82) in those receiving Dmab (n = 1204) compared with no treatment [73]. These publications reveal to some extent contradictory data on potential anticancer effects of adjuvant Dmab in post-MP women with HR + EBC. Therefore, the use of Dmab for reducing recurrence cannot be recommended currently.

## 4 Evaluation of fracture risk and indication to initiate anti-resorptive therapy

**Fig. 3:**
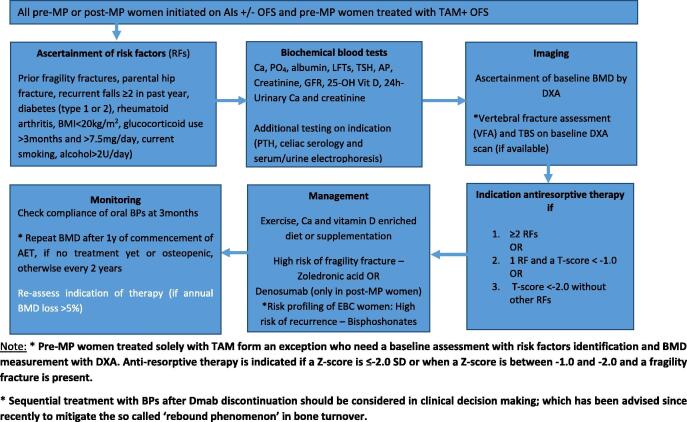
**Management algorithm for EBC women on adjuvant endocrine therapy adapted from Hadji *et al*.**[11]**.***EBC, early breast cancer; AIs, aromatase inhibitors; OFS, ovarian function suppression; BMI, body mass index; Ca, Calcium; PO_4_, phosphate; LFTs, liver function tests; RFs, risk factors;* RFTs, renal function tests; TSH, thyroid stimulating hormone. FRAX is not validated for women < 40 years of age.

Various screening and treatment algorithms have been published for monitoring bone health during endocrine therapy [11], [80], [81]. Nevertheless, multiple retrospective and prospective clinical studies observed a suboptimal real-world bone health care in breast cancer patients receiving AET, which led to under treatment with anti-resorptive therapy [82], [83]. Fig. 3 provides an adapted algorithm for optimal management of bone health in EBC women originally provided in Hadji *et al.* [11].

It is crucial that all women initiating AIs and/or OFS have a tailored medical history and examination, a baseline dual energy X-ray absorptiometry scan (DXA) and biochemical testing for ruling out additional secondary causes of osteoporosis.

Vertebral fracture assessment (VFA) along with DXA (or, if unavailable, conventional spine X-rays in selected cases) could be a part of screening or follow-up in all post-MP and osteopenic pre-MP women initiated on AET. Indeed, multiple cross-sectional studies reported a higher prevalence of morphometric vertebral fractures (VFs) both before (~20%) and during endocrine therapy (~31%) [84], [85] including a recent study by Pederseni *et al*. In this study, a higher number of VFs was found within AI-treated subjects, even in those with normal BMD (every 1 out of 5 women) [86]. However, an overestimation of VFs could not be excluded because of inclusion of Genant’s grade 1 fractures.

Higher prevalence of fractures in AET users even with normal BMD could be partly explained by deterioration of bone quality. A surrogate of bone quality-Trabecular Bone Score, a novel 2D microarchitecture assessment derived from DXA-images, was reported to be decreased in both pre- and post-MP women on AET at 24 months [37], [38]. Similarly, TBS also improved in the group concurrently treated with ZOL independent of BMD confirming that TBS reflects bone properties other than BMD [87], [88], [89]. Extrapolation of these findings suggests that using TBS and BMD jointly could translate into better prediction of fracture risk in AET users but the evidence is lacking in this regard.

The conventional fracture risk assessment tools such as FRAX® are not designed to assess fracture risk in women initiated on endocrine therapy. However, a common clinical practice in EBC women is to mark the option “secondary osteoporosis”, only operational in the absence of BMD*,* in the FRAX® tool to assess the fracture risk which may theoretically lead to fracture underestimation. In contrast, two recent studies found an overestimation of 10-year fracture probability in AIs users if secondary osteoporosis alone is used in the FRAX® tool without BMD [90], [91]. In addition, FRAX® is not validated in women younger than 40 years old. In summary, the advice remains not to use FRAX® in these women.

Clinicians should emphasize the importance of exercise (resistance and aerobic training) in women with EBC, along with optimal intake of calcium and vitamin D. It is advisable to stop smoking, reduce alcohol consumption and optimize daily dairy intake. Any medication that impairs bone health should be changed, if possible. In the opinion of the authors based on recent literature and current ESMO guidelines [71], every woman (pre- or post-MP) initiating on AIs and/or OFS should undergo fracture risk assessment using conventional risk factors and BMD measurement with DXA and VFA. Anti-resorptive therapy should be considered in all women with a BMD T-score < -2.0 SD or ≥ 2 risk factors including a BMD T-score < -1.0. Clinical risk factors known to increase fracture riskinclude prior fragility fractures, parental history of hip fracture, diabetes (type 1 or 2), BMI < 20 kg/m^2^, rheumatoid arthritis, history of recurrent falls ≥ 2 in the past year, glucocorticoid use > 3 months and > 7.5 mg daily, current smoking and alcohol > 2U/day.. A stringent BMD T-score less than −2.0 SD is recommended to initiate treatment in these women as besides bone mass, bone quality is also impaired after AET which leads to increased fracture risk irrespective of baseline BMD [68].

In pre-MP women treated solely with TAM, there is consistent but weak evidence on BMD loss and increased fracture risk based on small-scale RCTs and case-control studies but a lack of data on anti-resorptive therapy to guide treatment. Based on expert opinion, we propose that these women should be offered a baseline fracture risk assessment with evaluation of risk factors and BMD measurement with DXA and VFA. Anti-resorptive therapy can be considered if a Z-score is ≤ -2.0 SD or if a Z-score is between −1.0 and −2.0 and fragility fractures have been reported [92]. Still, dedicated RCTs with a long follow-up are needed in pre-MP women on TAM while it will remain difficult to precisely evaluate anti-fracture benefits in these women due to low fracture incidence.

Regarding the choice of anti-resorptive therapy in pre-MP women, there is now strong evidence for the use of adjuvant ZOL (4 mg, every 6 months - Q6M) for prevention of BMD loss (ABCSG-12/AZURE) with numerous studies reporting BMD loss in pre-MP women and some weak and conflicting evidence on the prevention of breast cancer recurrence (ABCSG-12/HOBOE/AZURE) in those treated with AIs/OFS. It is important to state that there may be partial recovery of BMD after termination of AET due to resumption of menstruation. Effective contraception is needed at initiation of BPs due to potential of harm to the fetus. Momentarily, we have no data on oral BPs and Dmab. Therefore, future studies focusing on bone health in pre-MP women should focus on fractures as primary outcome and use of adjuvant anti-resorptives to prevent fractures.

With regard to the choice of anti-resorptive therapy in post-MP women, Dmab (60 mg, Q6M) has shown the strongest evidence and remains the first choice for fracture prevention in women with low risk EBC even though new data for ZOL also suggest fracture reduction albeit as a secondary outcome during longer follow-up [50]. A recent network *meta*-analysis also confirms these inferences [93]. With respect to breast cancer survival benefits along with bone protection, oral or intravenous BPs remain the first choice. While evidence for ZOL (4 mg, Q6M) is still most robust, ibandronate and clodronate have not been proven inferior. At the same time, new RCTs could not establish breast cancer survival benefits of Dmab (ABCSG-18/D-CARE). Future studies should compare lower doses and longer interval (e.g., ZOL 5 mg, Q12M) between anti-resorptive administration in this subgroup.

With respect to follow-up, DXA should be repeated 1 year after AIs initiation if no anti-resorptive therapy is initiated and every 2 years if commenced on anti-resorptive therapy. An annual BMD loss of > 5% is an indication for re-assessment regarding anti-resorptive therapy.

It is important to point out that adjuvant Dmab should never be stopped promptly. A sequential treatment with BPs should be undertaken after Dmab discontinuation to mitigate the so called ‘rebound phenomenon’ in bone turnover that has been associated with a rapid loss of BMD gain and a risk, although rare, of developing multiple vertebral fractures [94], [95]. The recommended duration of anti-resorptive therapy should be oriented to the duration of endocrine therapy and absolute fracture risk. If extended adjuvant endocrine therapy after 5 years is taken into consideration, an individualized risk-to-benefit evaluation of fracture risk versus potential side effects with longer therapy is needed.

Frequent dosing regimens and gastro-intestinal side-effects of oral BPs have been associated with low compliance rates [96]. A frequently reported side-effect of intravenous ZOL is self-limiting flu-like complaints in the first week after infusion. Side-effects such as osteonecrosis of the jaw (ONJ) and atypical femur fracture (AFF) are rare but potential serious complications of both BPs and Dmab depending on their dose and treatment duration[97].

## 5 Conclusion and future prospects

Bone loss and fracture prevention need attention in EBC women receiving endocrine therapy as breast cancer is becoming a chronic disease due to improved prognosis. Conventionally, clinical risk factors and BMD were used to evaluate fracture risk, but the use of VFA and bone quality by means of TBS might additionally help to identify those with higher fracture risk independent of BMD. FRAX® use can still not be recommended based on the weak current evidence. Lifestyle modifications with inclusion of exercise should be promoted in all women on AET. In pre-MP women, intravenous zoledronate is the only drug reported to prevent bone loss in EBC. . In post-MP women, the choice of anti-resorptive may differ depending on the treatment target. Denosumab is preferred when fracture prevention is a major concern with low breast cancer recurrence risk, while the need for sequential treatment after denosumab termination due to risk of the rebound effect should be considered in clinical decision making. Bisphosphonates are preferred when disease recurrence prevention is a major concern in high risk breast cancer women along with bone health, while denosumab failed to show a decline in breast cancer recurrence..

In addition to the established risk factors for bone health, breast cancer specific risk factors such as obesity, adjuvant and neo-adjuvant chemo- and radiotherapy are important topics for future research. Based on current evidence, there are still no convincing data on routine use of bone turnover markers in EBC women started on AI therapy and during follow-up. Use of imaging techniques, such as high resolution peripheral quantitative computed tomography (HR-pQCT) are currently being evaluated and have potential implications for clinical practice in the future [98].

The use of adjuvant anti-resorptive therapy, particularly oral BPs and Dmab, in pre-MP women on AET with respect to fracture prevention as a primary outcome is an area that still merits more research. Whether Dmab for fracture prevention in post-MP women on AIs should be started irrespective of their baseline BMD status warrants more evidence. In the current setting, optimal dose and interval of denosumab in non-metastatic breast cancer (60 mg, 6monthly vs. 120 mg, every month/every 3 months) requires a consensus. Similarly, future head-to-head comparisons of 6 monthly 4 mg ZOL vs. once yearly 5 mg ZOL regimens in AIs users could have financial implications for clinical practice.

## Funding

This research did not receive any specific grant from any funding agency in the public, commercial or not-for-profit sector.

## Declaration of Competing Interest

The authors declare that they have no known competing financial interests or personal relationships that could have appeared to influence the work reported in this paper.

## References

[1] Ginsburg O., Bray F., Coleman M.P., Vanderpuye V., Eniu A., Kotha S.R. (2017). The global burden of women's cancers: a grand challenge in global health. Lancet.

[2] Winters S., Martin C., Murphy D., Shokar N.K. (2017). Breast Cancer Epidemiology, Prevention, and Screening. Prog. Mol. Biol. Transl. Sci..

[3] Pierce S.M., Recht A., Lingos T.I., Abner A., Vicini F., Silver B. (1992). Long-term radiation complications following conservative surgery (CS) and radiation therapy (RT) in patients with early stage breast cancer. Int. J. Radiat. Oncol. Biol. Phys..

[4] Hain B.A., Xu H., Wilcox J.R., Mutua D., Waning D.L. (2019). Chemotherapy-induced loss of bone and muscle mass in a mouse model of breast cancer bone metastases and cachexia. JCSM Rapid Commun..

[5] van Leeuwen B.L., Hartel R.M., Jansen H.W., Kamps W.A., Hoekstra H.J. (2003). The effect of chemotherapy on the morphology of the growth plate and metaphysis of the growing skeleton. Eur. J. Surg. Oncol..

[6] Stumpf U., Kostev K., Kyvernitakis J., Böcker W., Hadji P. (2019). Incidence of fractures in young women with breast cancer - a retrospective cohort study. J Bone Oncol..

[7] Rao RD, Cobleigh MA. Adjuvant endocrine therapy for breast cancer. Oncology (Williston Park). 2012;26(6):541-7, 50, 52 passim. 22870539

[8] Shah R., O'Regan R.M. (2018). Adjuvant Endocrine Therapy. Cancer Treat. Res..

[9] Kyvernitakis I., Kostev K., Hadji P. (2018). The tamoxifen paradox-influence of adjuvant tamoxifen on fracture risk in pre- and postmenopausal women with breast cancer. Osteoporos. Int..

[10] Stumpf U., Kostev K., Kyvernitakis J., Bocker W., Hadji P. (2019). Incidence of fractures in young women with breast cancer - a retrospective cohort study. J Bone Oncol..

[11] Hadji P., Aapro M.S., Body J.J., Gnant M., Brandi M.L., Reginster J.Y. (2017). Management of Aromatase Inhibitor-Associated Bone Loss (AIBL) in postmenopausal women with hormone sensitive breast cancer: Joint position statement of the IOF, CABS, ECTS, IEG, ESCEO IMS, and SIOG. J Bone Oncol..

[12] Gnant M., Mlineritsch B., Luschin-Ebengreuth G., Kainberger F., Kassmann H., Piswanger-Solkner J.C. (2008). Adjuvant endocrine therapy plus zoledronic acid in premenopausal women with early-stage breast cancer: 5-year follow-up of the ABCSG-12 bone-mineral density substudy. Lancet Oncol..

[13] Sverrisdóttir A., Fornander T., Jacobsson H., von Schoultz E., Rutqvist L.E. (2004). Bone mineral density among premenopausal women with early breast cancer in a randomized trial of adjuvant endocrine therapy. J. Clin. Oncol..

[14] Powles T.J., Hickish T., Kanis J.A., Tidy A., Ashley S. (1996). Effect of tamoxifen on bone mineral density measured by dual-energy x-ray absorptiometry in healthy premenopausal and postmenopausal women. J. Clin. Oncol..

[15] Fogelman I., Blake G.M., Blamey R., Palmer M., Sauerbrei W., Schumacher M. (2003). Bone mineral density in premenopausal women treated for node-positive early breast cancer with 2 years of goserelin or 6 months of cyclophosphamide, methotrexate and 5-fluorouracil (CMF). Osteoporos. Int..

[16] Kalder M., Kyvernitakis I., Albert U.S., Baier-Ebert M., Hadji P. (2015). Effects of zoledronic acid versus placebo on bone mineral density and bone texture analysis assessed by the trabecular bone score in premenopausal women with breast cancer treatment-induced bone loss: results of the ProBONE II substudy. Osteoporos. Int..

[17] Catalano A., Gaudio A., Agostino R.M., Morabito N., Bellone F., Lasco A. (2019). Trabecular bone score and quantitative ultrasound measurements in the assessment of bone health in breast cancer survivors assuming aromatase inhibitors. J. Endocrinol. Invest..

[18] Gnant M., Mlineritsch B., Stoeger H., Luschin-Ebengreuth G., Heck D., Menzel C. (2011). Adjuvant endocrine therapy plus zoledronic acid in premenopausal women with early-stage breast cancer: 62-month follow-up from the ABCSG-12 randomised trial. Lancet Oncol..

[19] Francis P.A., Pagani O., Fleming G.F., Walley B.A., Colleoni M., Láng I. (2018). Tailoring adjuvant endocrine therapy for premenopausal breast cancer. New Engl J Med..

[20] Perrone F., De Laurentiis M., De Placido S., Orditura M., Cinieri S., Riccardi F. (2019). Adjuvant zoledronic acid and letrozole plus ovarian function suppression in premenopausal breast cancer: HOBOE phase 3 randomised trial. Eur. J. Cancer.

[21] Kyvernitakis I., Kostev K., Hadji P. (2018). The tamoxifen paradox—influence of adjuvant tamoxifen on fracture risk in pre- and postmenopausal women with breast cancer. Osteoporosis Int..

[22] Heshmati H.M., Khosla S., Robins S.P., O'Fallon W.M., Melton L.J., Riggs B.L. (2002). Role of low levels of endogenous estrogen in regulation of bone resorption in late postmenopausal women. J. Bone Miner. Res..

[23] Coleman R.E., Banks L.M., Girgis S.I., Kilburn L.S., Vrdoljak E., Fox J. (2007). Skeletal effects of exemestane on bone-mineral density, bone biomarkers, and fracture incidence in postmenopausal women with early breast cancer participating in the Intergroup Exemestane Study (IES): a randomised controlled study. Lancet Oncol..

[24] Aihara T., Suemasu K., Takei H., Hozumi Y., Takehara M., Saito T. (2010). Effects of exemestane, anastrozole and tamoxifen on bone mineral density and bone turnover markers in postmenopausal early breast cancer patients: results of N-SAS BC 04, the TEAM Japan substudy. Oncology..

[25] Hadji P., Ziller M., Kieback D.G., Menschik T., Kalder M., Kuck J. (2009). The effect of exemestane or tamoxifen on markers of bone turnover: results of a German sub-study of the Tamoxifen Exemestane Adjuvant Multicentre (TEAM) trial. Breast..

[26] Eastell R., Adams J., Clack G., Howell A., Cuzick J., Mackey J. (2011). Long-term effects of anastrozole on bone mineral density: 7-year results from the ATAC trial. Ann. Oncol..

[27] Zaman K., Thurlimann B., Huober J., Schonenberger A., Pagani O., Luthi J. (2012). Bone mineral density in breast cancer patients treated with adjuvant letrozole, tamoxifen, or sequences of letrozole and tamoxifen in the BIG 1–98 study (SAKK 21/07). Ann. Oncol..

[28] Decensi A., Sun Z., Guerrieri-Gonzaga A., Thurlimann B., McIntosh C., Tondini C. (2014). Bone mineral density and circulating biomarkers in the BIG 1–98 trial comparing adjuvant letrozole, tamoxifen and their sequences. Breast Cancer Res. Treat..

[29] Jakesz R., Jonat W., Gnant M., Mittlboeck M., Greil R., Tausch C. (2005). Switching of postmenopausal women with endocrine-responsive early breast cancer to anastrozole after 2 years' adjuvant tamoxifen: combined results of ABCSG trial 8 and ARNO 95 trial. Lancet.

[30] Perez E.A., Josse R.G., Pritchard K.I., Ingle J.N., Martino S., Findlay B.P. (2006). Effect of letrozole versus placebo on bone mineral density in women with primary breast cancer completing 5 or more years of adjuvant tamoxifen: a companion study to NCIC CTG MA.17. J. Clin. Oncol..

[31] Amir E., Seruga B., Niraula S., Carlsson L., Ocana A. (2011). Toxicity of adjuvant endocrine therapy in postmenopausal breast cancer patients: a systematic review and meta-analysis. J. Natl Cancer Inst..

[32] Tseng O.L., Spinelli J.J., Gotay C.C., Ho W.Y., McBride M.L., Dawes M.G. (2018). Aromatase inhibitors are associated with a higher fracture risk than tamoxifen: a systematic review and meta-analysis. Ther Adv Musculoskelet Dis..

[33] Pineda-Moncusí M., Servitja S., Tusquets I., Diez-Perez A., Rial A., Cos M.L. (2019). Assessment of early therapy discontinuation and health-related quality of life in breast cancer patients treated with aromatase inhibitors: B-ABLE cohort study. Breast Cancer Res. Treat..

[34] van Hellemond I.E.G., Geurts S.M.E., Tjan-Heijnen V.C.G. (2018). Current Status of Extended Adjuvant Endocrine Therapy in Early Stage Breast Cancer. Curr. Treat. Options Oncol..

[35] van Hellemond I.E.G., Smorenburg C.H., Peer P.G.M., Swinkels A.C.P., Seynaeve C.M., van der Sangen M.J.C. (2019). Assessment and management of bone health in women with early breast cancer receiving endocrine treatment in the DATA study. Int. J. Cancer.

[36] Van Hellemond IE, Smorenburg CH, Peer PG, Swinkels AC, Seynaeve CM, Van Der Sangen MJ, et al. No impact of osteoporosis or bisphosphonate use for osteoporosis on breast cancer outcome: a sub-study of the DATA trial. Cancer research. 2019;79(4).

[37] Mamounas E.P., Bandos H., Lembersky B.C., Jeong J.H., Geyer C.E., Rastogi P. (2019). Use of letrozole after aromatase inhibitor-based therapy in postmenopausal breast cancer (NRG Oncology/NSABP B-42): a randomised, double-blind, placebo-controlled, phase 3 trial. Lancet Oncol..

[38] Goldvaser H., Barnes T.A., Seruga B., Cescon D.W., Ocaña A., Ribnikar D. (2018). Toxicity of extended adjuvant therapy with aromatase inhibitors in early breast cancer: A systematic review and meta-analysis. J. Natl Cancer Inst..

[39] Fornusek C.P., Kilbreath S.L. (2017). Exercise for improving bone health in women treated for stages I-III breast cancer: a systematic review and meta-analyses. J. Cancer Surviv..

[40] Saarto T., Sievanen H., Kellokumpu-Lehtinen P., Nikander R., Vehmanen L., Huovinen R. (2012). Effect of supervised and home exercise training on bone mineral density among breast cancer patients. A 12-month randomised controlled trial. Osteoporos. Int..

[41] Thomas G.A., Cartmel B., Harrigan M., Fiellin M., Capozza S., Zhou Y. (2017). The effect of exercise on body composition and bone mineral density in breast cancer survivors taking aromatase inhibitors. Obesity..

[42] de Paulo T.R.S., Winters-Stone K.M., Viezel J., Rossi F.E., Simões R.R., Tosello G. (2018). Effects of resistance plus aerobic training on body composition and metabolic markers in older breast cancer survivors undergoing aromatase inhibitor therapy. Exp. Gerontol..

[43] Peppone L.J., Ling M., Huston A.J., Reid M.E., Janelsins M.C., Puzas J.E. (2018). The effects of high-dose calcitriol and individualized exercise on bone metabolism in breast cancer survivors on hormonal therapy: a phase II feasibility trial. Supportive Care Cancer..

[44] Casla S., Lopez-Tarruella S., Jerez Y., Marquez-Rodas I., Galvao D.A., Newton R.U. (2015). Supervised physical exercise improves VO2max, quality of life, and health in early stage breast cancer patients: a randomized controlled trial. Breast Cancer Res. Treat..

[45] Ligibel J. (2012). Lifestyle factors in cancer survivorship. J. Clin. Oncol..

[46] Watanabe A., Yoneyama S., Nakajima M., Sato N., Takao-Kawabata R., Isogai Y. (2012). Osteosarcoma in Sprague-Dawley rats after long-term treatment with teriparatide (human parathyroid hormone (1–34)). J. Toxicol. Sci..

[47] Jolette J., Attalla B., Varela A., Long G.G., Mellal N., Trimm S. (2017). Comparing the incidence of bone tumors in rats chronically exposed to the selective PTH type 1 receptor agonist abaloparatide or PTH(1–34). Regul. Toxicol. Pharm..

[48] Nuzzo F., Gallo C., Lastoria S., Di Maio M., Piccirillo M.C., Gravina A. (2012). Bone effect of adjuvant tamoxifen, letrozole or letrozole plus zoledronic acid in early-stage breast cancer: the randomized phase 3 HOBOE study. Ann. Oncol..

[49] Kyvernitakis I., Kann P.H., Thomasius F., Hars O., Hadji P. (2018). Prevention of breast cancer treatment-induced bone loss in premenopausal women treated with zoledronic acid: Final 5-year results from the randomized, double-blind, placebo-controlled ProBONE II trial. Bone.

[50] Wilson C., Bell R., Hinsley S., Marshall H., Brown J., Cameron D. (2018). Adjuvant zoledronic acid reduces fractures in breast cancer patients; an AZURE (BIG 01/04) study. Eur. J. Cancer.

[51] Hines S.L., Mincey B., Dentchev T., Sloan J.A., Perez E.A., Johnson D.B. (2009). Immediate versus delayed zoledronic acid for prevention of bone loss in postmenopausal women with breast cancer starting letrozole after tamoxifen-N03CC. Breast Cancer Res. Treat..

[52] Llombart A., Frassoldati A., Paija O., Sleeboom H.P., Jerusalem G., Mebis J. (2012). Immediate Administration of Zoledronic Acid Reduces Aromatase Inhibitor-Associated Bone Loss in Postmenopausal Women With Early Breast Cancer: 12-month analysis of the E-ZO-FAST trial. Clin Breast Cancer..

[53] Eidtmann H., de Boer R., Bundred N., Llombart-Cussac A., Davidson N., Neven P. (2010). Efficacy of zoledronic acid in postmenopausal women with early breast cancer receiving adjuvant letrozole: 36-month results of the ZO-FAST Study. Ann. Oncol..

[54] Coleman R., de Boer R., Eidtmann H., Llombart A., Davidson N., Neven P. (2013). Zoledronic acid (zoledronate) for postmenopausal women with early breast cancer receiving adjuvant letrozole (ZO-FAST study): final 60-month results. Ann. Oncol..

[55] Brufsky A. (2006). Management of cancer-treatment-induced bone loss in postmenopausal women undergoing adjuvant breast cancer therapy: a Z-FAST update. Semin. Oncol..

[56] Gnant M. (2011). Zoledronic acid in breast cancer: latest findings and interpretations. Ther Adv Med Oncol..

[57] Van Poznak C., Hannon R.A., Mackey J.R., Campone M., Apffelstaedt J.P., Clack G. (2010). Prevention of aromatase inhibitor-induced bone loss using risedronate: the SABRE trial. J. Clin. Oncol..

[58] Greenspan S.L., Vujevich K.T., Brufsky A., Lembersky B.C., van Londen G.J., Jankowitz R.C. (2015). Prevention of bone loss with risedronate in breast cancer survivors: a randomized, controlled clinical trial. Osteoporos. Int..

[59] Lester J.E., Dodwell D., Purohit O.P., Gutcher S.A., Ellis S.P., Thorpe R. (2008). Prevention of anastrozole-induced bone loss with monthly oral ibandronate during adjuvant aromatase inhibitor therapy for breast cancer. Clin. Cancer Res..

[60] Sestak I., Singh S., Cuzick J., Blake G.M., Patel R., Gossiel F. (2014). Changes in bone mineral density at 3 years in postmenopausal women receiving anastrozole and risedronate in the IBIS-II bone substudy: an international, double-blind, randomised, placebo-controlled trial. Lancet Oncol..

[61] Rhee Y., Song K., Park S., Park H.S., Lim S.K., Park B.W. (2013). Efficacy of a combined alendronate and calcitriol agent (Maxmarvil(R)) in Korean postmenopausal women with early breast cancer receiving aromatase inhibitor: a double-blind, randomized, placebo-controlled study. Endocr. J..

[62] Pineda-Moncusí M., Garcia-Giralt N., Diez-Perez A., Servitja S., Tusquets I., Prieto-Alhambra D. (2019). Increased Fracture Risk in Women Treated With Aromatase Inhibitors Versus Tamoxifen: Beneficial Effect of Bisphosphonates. J. Bone Miner. Res..

[63] Brufsky A.M., Bosserman L.D., Caradonna R.R., Haley B.B., Jones C.M., Moore H.C. (2009). Zoledronic acid effectively prevents aromatase inhibitor-associated bone loss in postmenopausal women with early breast cancer receiving adjuvant letrozole: Z-FAST study 36-month follow-up results. Clin Breast Cancer..

[64] Bouxsein M.L., Eastell R., Lui L.Y., Wu L.A., de Papp A.E., Grauer A. (2019). Change in Bone Density and Reduction in Fracture Risk: A Meta-Regression of Published Trials. J. Bone Miner. Res..

[65] Nakatsukasa K., Koyama H., Ouchi Y., Sakaguchi K., Fujita Y., Matsuda T. (2019). Effects of denosumab on bone mineral density in Japanese women with osteoporosis treated with aromatase inhibitors for breast cancer. J. Bone Miner. Metab..

[66] Nakatsukasa K., Koyama H., Ouchi Y., Sakaguchi K., Fujita Y., Matsuda T. (2018). Effect of denosumab administration on low bone mineral density (T-score −1.0 to −2.5) in postmenopausal Japanese women receiving adjuvant aromatase inhibitors for non-metastatic breast cancer. J. Bone Miner. Metab..

[67] Nakatsukasa K., Koyama H., Ouchi Y., Ono H., Sakaguchi K., Matsuda T. (2019). Effect of denosumab on low bone mineral density in postmenopausal Japanese women receiving adjuvant aromatase inhibitors for non-metastatic breast cancer: 24-month results. Breast Cancer..

[68] Gnant M., Pfeiler G., Dubsky P.C., Hubalek M., Greil R., Jakesz R. (2015). Adjuvant denosumab in breast cancer (ABCSG-18): a multicentre, randomised, double-blind, placebo-controlled trial. Lancet.

[69] Croucher P.I., McDonald M.M., Martin T.J. (2016). Bone metastasis: the importance of the neighbourhood. Nat. Rev. Cancer.

[70] (2015). Lancet.

[71] Coleman R., Hadji P., Body J.J., Santini D., Chow E., Terpos E. (2020). Bone health in cancer: ESMO Clinical Practice Guidelines. Ann. Oncol..

[72] Early Breast Cancer Trialists' Collaborative G. Adjuvant bisphosphonate treatment in early breast cancer: meta-analyses of individual patient data from randomised trials. Lancet. 2015, 386(10001):1353-61. 10.1016/S0140-6736(15)60908-4 26211824

[73] Suarez-Almazor M.E., Herrera R., Lei X., Chavez-MacGregor M., Zhao H., Giordano S.H. (2020). Survival in older women with early stage breast cancer receiving low-dose bisphosphonates or denosumab. Cancer.

[74] Gralow J.R., Barlow W.E., Paterson A.H.G., Miao J.L., Lew D.L., Stopeck A.T. (2019). Phase III randomized trial of bisphosphonates as adjuvant therapy in breast cancer: S0307. J. Natl Cancer Inst..

[75] Rennert G., Pinchev M., Gronich N., Saliba W., Flugelman A., Lavi I. (2017). Oral Bisphosphonates and Improved Survival of Breast Cancer. Clin. Cancer Res..

[76] Bouvard B., Chatelais J., Soulié P., Hoppé E., Saulnier P., Capitain O. (2018). Osteoporosis treatment and 10 years' oestrogen receptor+ breast cancer outcome in postmenopausal women treated with aromatase inhibitors. Eur. J. Cancer.

[77] Gnant M., Pfeiler G., Steger G.G., Egle D., Greil R., Fitzal F. (2019). Adjuvant denosumab in postmenopausal patients with hormone receptor-positive breast cancer (ABCSG-18): disease-free survival results from a randomised, double-blind, placebo-controlled, phase 3 trial. Lancet Oncol..

[78] Gnant M., Pfeiler G., Frantal S. (2019). Denosumab in early-stage breast cancer – Authors' reply. Lancet Oncol..

[79] Coleman R., Finkelstein D.M., Barrios C., Martin M., Iwata H., Hegg R. (2019). Adjuvant denosumab in early breast cancer (D-CARE): an international, multicentre, randomised, controlled, phase 3 trial. Lancet Oncol..

[80] Grossmann M., Ramchand S.K., Milat F., Vincent A., Lim E., Kotowicz M.A. (2018). Assessment and management of bone health in women with oestrogen receptor-positive breast cancer receiving endocrine therapy: Position statement of the Endocrine Society of Australia, the Australian and New Zealand Bone & Mineral Society, the Australasian Menopause Society and the Clinical Oncology Society of Australia. Clin. Endocrinol..

[81] Rachner T.D., Coleman R., Hadji P., Hofbauer L.C. (2018). Bone health during endocrine therapy for cancer. Lancet Diabetes Endocrinol..

[82] Hamood R., Hamood H., Merhasin I., Keinan-Boker L. (2019). Hormone therapy and osteoporosis in breast cancer survivors: assessment of risk and adherence to screening recommendations. Osteoporosis Int..

[83] Tremblay D., Patel V., Fifer K.M., Caro J., Kolodka O., Mandelli J. (2018). Management of bone health in postmenopausal women on aromatase inhibitors (AIs): a single health care system experience. Supportive Care Cancer..

[84] Villa P., Lassandro A.P., Amar I.D., Vacca L., Moruzzi M.C., Ferrandina G. (2016). Impact of aromatase inhibitor treatment on vertebral morphology and bone mineral density in postmenopausal women with breast cancer. Menopause..

[85] Bouvard B., Hoppe E., Soulie P., Georgin-Mege M., Jadaud E., Abadie-Lacourtoisie S. (2012). High prevalence of vertebral fractures in women with breast cancer starting aromatase inhibitor therapy. Ann. Oncol..

[86] Pedersini R., Monteverdi S., Mazziotti G., Amoroso V., Roca E., Maffezzoni F. (2017). Morphometric vertebral fractures in breast cancer patients treated with adjuvant aromatase inhibitor therapy: A cross-sectional study. Bone.

[87] Hong A.R., Kim J.H., Lee K.H., Kim T.Y., Im S.A., Kim T.Y. (2017). Long-term effect of aromatase inhibitors on bone microarchitecture and macroarchitecture in non-osteoporotic postmenopausal women with breast cancer. Osteoporosis Int..

[88] Rodriguez-Sanz M, Pineda-Moncusi M, Garcia-Giralt N, Servitja S, Martos T, Blanch-Rubio J, et al. TBS variation in breast cancer women completing AI-therapy: a prospective study of the B-able cohort. Journal of bone and mineral research Conference: 2016 annual meeting of the american society for bone and mineral research, ASBMR 2016 United states. 2017;31(Supplement 1) (no pagination).

[89] Mariotti V., Page D.B., Davydov O., Hans D., Hudis C.A., Patil S. (2017). Assessing fracture risk in early stage breast cancer patients treated with aromatase-inhibitors: An enhanced screening approach incorporating trabecular bone score. J Bone Oncol..

[90] Prawiradilaga R.S., Gunmalm V., Lund-Jacobsen T., Helge E.W., Brøns C., Andersson M. (2018). FRAX Calculated without BMD Resulting in a Higher Fracture Risk Than That Calculated with BMD in Women with Early Breast Cancer. J Osteoporosis..

[91] Leslie W.D., Morin S.N., Lix L.M., Niraula S., McCloskey E.V., Johansson H. (2019). Performance of FRAX in Women with Breast Cancer Initiating Aromatase Inhibitor Therapy: A Registry-Based Cohort Study. J. Bone Miner. Res..

[92] Pepe J., Body J.J., Hadji P., McCloskey E., Meier C., Obermayer-Pietsch B. (2020). Osteoporosis in Premenopausal Women: A Clinical Narrative Review by the ECTS and the IOF. J. Clin. Endocrinol. Metab..

[93] Miyashita H., Satoi S., Kuno T., Cruz C., Malamud S., Kim S.M. (2020). Bone modifying agents for bone loss in patients with aromatase inhibitor as adjuvant treatment for breast cancer; insights from a network meta-analysis. Breast Cancer Res. Treat..

[94] Gonzalez-Rodriguez E., Aubry-Rozier B., Stoll D., Zaman K., Lamy O. (2019). Sixty spontaneous vertebral fractures after denosumab discontinuation in 15 women with early-stage breast cancer under aromatase inhibitors. Breast Cancer Res. Treat..

[95] Tsourdi E., Zillikens M.C., Meier C., Body J.J., Gonzalez Rodriguez E., Anastasilakis A.D. (2020). Fracture risk and management of discontinuation of denosumab therapy: a systematic review and position statement by ECTS. J. Clin. Endocrinol. Metab..

[96] Carbonell-Abella C., Pages-Castella A., Javaid M.K., Nogues X., Farmer A.J., Cooper C. (2015). Early (1-year) Discontinuation of Different Anti-osteoporosis Medications Compared: A Population-Based Cohort Study. Calcif. Tissue Int..

[97] Adler R.A., El-Hajj Fuleihan G., Bauer D.C., Camacho P.M., Clarke B.L., Clines G.A. (2016). Managing Osteoporosis in Patients on Long-Term Bisphosphonate Treatment: Report of a Task Force of the American Society for Bone and Mineral Research. J. Bone Miner. Res..

[98] Hopson M.B., Onishi M., Awad D., Buono D., Maurer M., Crew K.D. (2020). Prospective Study Evaluating Changes in Bone Quality in Premenopausal Women With Breast Cancer Undergoing Adjuvant Chemotherapy. Clin Breast Cancer.

[99] Livi L., Scotti V., Desideri I., Saieva C., Cecchini S., Francolini G. (2019). Phase 2 placebo-controlled, single-blind trial to evaluate the impact of oral ibandronate on bone mineral density in osteopenic breast cancer patients receiving adjuvant aromatase inhibitors: 5-year results of the single-centre BONADIUV trial. Eur. J. Cancer.

[100] Coleman R.E., Collinson M., Gregory W., Marshall H., Bell R., Dodwell D. (2018). Benefits and risks of adjuvant treatment with zoledronic acid in stage II/III breast cancer. 10 years follow-up of the AZURE randomized clinical trial (BIG 01/04). J Bone Oncol..

[101] Santa-Maria C.A., Bardia A., Blackford A.L., Snyder C., Connolly R.M., Fetting J.H. (2018). A phase II study evaluating the efficacy of zoledronic acid in prevention of aromatase inhibitor-associated musculoskeletal symptoms: the ZAP trial. Breast Cancer Res. Treat..

[102] Sestak I., Blake G.M., Patel R., Coleman R.E., Cuzick J., Eastell R. (2019). Comparison of risedronate versus placebo in preventing anastrozole-induced bone loss in women at high risk of developing breast cancer with osteopenia. Bone.

[103] Monda V., Lupoli G.A., Messina G., Peluso R., Panico A., Villano I. (2017). Improvement of bone physiology and life quality due to association of risedronate and anastrozole. Front. Pharmacol..

