# Supplementary material for: Updated guidance on the management of cancer treatment-induced bone loss (CTIBL) in pre- and postmenopausal women with early-stage breast cancer
Source: J Bone Oncol. 2021 Mar 18;28:100355. doi: 10.1016/j.jbo.2021.100355 (PMC8080519; doi:10.1016/j.jbo.2021.100355)
Supplement: Supplementary data 1 [file mmc1.docx]

**Supplementary table 1. Categorization of included studies on adjuvant endocrine therapy (AET) and bone health from January 2017 to May/June 2020.**

| Classification of data on AET and bone health from January 2017 to May 2020 | | No. | Original studies, Reviews, Comments, Reply |
| --- | --- | --- | --- |
| Fractures / BMD / Bone Markers | | 27 | [1-27] |
| Bone quality (majority TBS, 1 QUS, 1 HRpQCT) | | 11 | [28-38] |
| Extended endocrine therapy and fractures | | 12 | [39-50] |
| Fracture risk assessment | | 12 | [38, 51-61] |
| Non-pharmacological | | 12 | [62-73] |
| Anti-resorptive therapy and bone loss/fracture prevention | BPs | 13 | [74-86] |
|  | Dmab | 16 | [87-102] |
| Anti-resoptive therapy and additional survival benefits | BPs | 10 | [103-112] |
|  | Dmab | 2 | [113-115] |
| Reviews on prevention of bone loss and/or its mangement in AET | | 30 | [116-144] |
| Total | | 144 |  |

**Supplementary document 1. Original terms used in the search engine to obtain the selected studies**

**(01-01-2017 till 30-05-2020).**

**“-----------------------------------------**

**embase.com**

('aromatase inhibitor'/de OR anastrozole/de OR exemestane/de OR letrozole/de ***OR*** ***(('hormonal therapy'/de OR 'cancer hormone therapy'/de) AND ('breast cancer'/exp OR 'breast tumor'/de))*** ***OR 'tamoxifen'/de*** OR ((aromatase NEAR/3 inhibitor*) OR anastrozol* OR exemestan* OR letrozol* ***OR ((hormon*-therap* OR endocrin*-therap*) NEAR/3 (breast* OR mamma*) NEAR/3 (cancer* OR neoplas* OR tumor* OR tumour*)) OR tamoxifen****):ab,ti,kw) AND ('bone health'/de OR 'fracture'/exp OR 'bone demineralization'/exp OR 'bone density'/de OR 'orthopedic software'/de OR 'bisphosphonic acid derivative'/exp OR 'denosumab'/de OR 'bone disease'/de OR 'bone atrophy'/de OR 'bone metabolism'/exp OR (((bone OR osseous) NEAR/3 (health OR demineral* OR mineral* OR densit* OR loss OR turnover* OR disease* OR metabol* OR mineral* OR atroph* OR absorpt* OR resorpt* OR degenerat*)) OR fracture* OR osteoporo* OR frax OR bisphosphon* OR denosumab* OR zoledron*):ab,ti,kw) AND ***[2016-2020]/py*** NOT ([animals]/lim NOT [humans]/lim) NOT ([Conference Abstract]/lim) ***AND [English]/lim***

**Medline ovid**

(Aromatase Inhibitors/ OR Anastrozole/ OR exemestane.nm. OR Letrozole/ ***OR (Hormone Replacement Therapy/ AND exp Breast Neoplasms/) OR Tamoxifen/*** OR ((aromatase ADJ3 inhibitor*) OR anastrozol* OR exemestan* OR letrozol* ***OR ((hormon*-therap* OR endocrin*-therap*) ADJ3 (breast* OR mamma*) ADJ3 (cancer* OR neoplas* OR tumor* OR tumour*)) OR tamoxifen****).ab,ti,kf.) AND (Fractures, Bone/ OR Bone Density/ OR exp Diphosphonates/ OR Denosumab/ OR Bone Diseases/ OR (((bone OR osseous) ADJ3 (health OR demineral* OR mineral* OR densit* OR loss OR turnover* OR disease* OR metabol* OR mineral* OR atroph* OR absorpt* OR resorpt* OR degenerat*)) OR fracture* OR osteoporo* OR frax OR bisphosphon* OR denosumab* OR zoledron*).ab,ti,kf.) AND ***(2016 OR 2017 OR 2018 OR 2019 OR 2020).yr.*** NOT (exp animals/ NOT humans/) NOT (news OR congres* OR abstract* OR book* OR chapter* OR dissertation abstract*).pt. ***AND English.lg.***

**Cochrane CENTRAL**

(((aromatase NEAR/3 inhibitor*) OR anastrozol* OR exemestan* OR letrozol* ***OR ((hormon* NEXT therap* OR endocrin* NEXT therap*) NEAR/3 (breast* OR mamma*) NEAR/3 (cancer* OR neoplas* OR tumor* OR tumour*)) OR tamoxifen****):ab,ti) AND ((((bone OR osseous) NEAR/3 (health OR demineral* OR mineral* OR densit* OR loss OR turnover* OR disease* OR metabol* OR mineral* OR atroph* OR absorpt* OR resorpt* OR degenerat*)) OR fracture* OR osteoporo* OR frax OR bisphosphon* OR denosumab* OR zoledron*):ab,ti)

**-----------------------------------------”**

**References:**

1. Bruyère O, Bergmann P, Cavalier E, Gielen E, Goemaere S, Kaufman JM, et al. Skeletal health in breast cancer survivors. Maturitas. 2017;105:78-82.

2. Ferreira Poloni P, Vespoli HDL, Almeida-Filho BDS, Bueloni-Dias F, Nahas-Neto J, Nahas EAP. Low bone mineral density is associated with breast cancer in postmenopausal women: a case–control study. Climacteric. 2017;20(5):491-7.

3. Francis PA, Fleming GF, Regan MM, Pagani O, Walley BA, Price KN, et al. Longterm follow-up of TEXT and SOFT trials of adjuvant endocrine therapies for premenopausal women with HR+ early breast cancer. Cancer research. 2017;77(4).

4. Khachidze N, Giorgadze E, Tsagareli M. ADJUVANT (HORMONAL) THERAPY AS A CAUSE OF BONE LOSS IN PATIENTS WITH BREAST CANCER (REVIEW OF LITERATURE). Georgian Med News. 2017(262):39-42.

5. Zikan V, Zimovjanova M, Michalska D, Raskova M, Petruzelka L. Changes in bone mineral density and biochemical markers of bone turnover in postmenopausal women with breast cancer initiating aromatase inhibitor therapy. Journal of bone and mineral research Conference: 2016 annual meeting of the american society for bone and mineral research, ASBMR 2016 United states. 2017;31(Supplement 1) (no pagination).

6. Axelsen CT, Jensen AB, Jakobsen EH, Bechmann T. Bone loss during neoadjuvant/adjuvant chemotherapy for early stage breast cancer: A retrospective cohort study. Mol Clin Oncol. 2018;8(6):767-72.

7. Kristensen B, Ejlertsen B, Jensen MB, Mouridsen HT. The occurrence of fractures after adjuvant treatment of breast cancer: a DBCG register study. Acta Oncol. 2018;57(1):141-5.

8. Kwan ML, Yao S, Laurent CA, Roh JM, Quesenberry CP, Kushi LH, et al. Changes in bone mineral density in women with breast cancer receiving aromatase inhibitor therapy. Breast Cancer Res Treat. 2018;168(2):523-30.

9. Kyvernitakis I, Kostev K, Hadji P. The tamoxifen paradox—influence of adjuvant tamoxifen on fracture risk in pre- and postmenopausal women with breast cancer. Osteoporosis Int. 2018;29(11):2557-64.

10. Neuner JM, Shi Y, Kong AL, Kamaraju S, Smith EC, Smallwood AJ, et al. Fractures in a nationwide population-based cohort of users of breast cancer hormonal therapy. J Cancer Surviv. 2018;12(2):268-75.

11. Ramin C, May BJ, Roden RBS, Orellana MM, Hogan BC, McCullough MS, et al. Evaluation of osteopenia and osteoporosis in younger breast cancer survivors compared with cancer-free women: a prospective cohort study. Breast Cancer Res. 2018;20(1):134.

12. Suskin J, Shapiro CL. Osteoporosis and musculoskeletal complications related to therapy of breast cancer Review. Gland surg. 2018;7(4):411-23.

13. Taxel P, Faircloth E, Idrees S, Van Poznak C. Cancer treatment-induced bone loss in women with breast cancer and men with prostate cancer. J Endocr Soc. 2018;2(7):574-88.

14. Thomasius F, Hadji P. Osteoporosis in patients with hormonal ablative therapy. Osteologie. 2018;27(1):14-9.

15. Tseng OL, Spinelli JJ, Gotay CC, Ho WY, McBride ML, Dawes MG. Aromatase inhibitors are associated with a higher fracture risk than tamoxifen: a systematic review and meta-analysis. Ther Adv Musculoskelet Dis. 2018;10(4):71-90.

16. Hung SC, Liao KF, Hung HC, Lin CL, Lee PC, Hung SJ, et al. Tamoxifen use correlates with increased risk of hip fractures in older women with breast cancer: A case–control study in Taiwan. Geriatr Gerontol Int. 2019;19(1):56-60.

17. Leslie WD, Morin SN, Lix LM, Niraula S, McCloskey EV, Johansson H, et al. Fracture Risk in Women with Breast Cancer Initiating Aromatase Inhibitor Therapy: A Registry-Based Cohort Study. Oncologist. 2019;24(11):1432-8.

18. Pineda-Moncusí M, Garcia-Giralt N, Diez-Perez A, Servitja S, Tusquets I, Prieto-Alhambra D, et al. Increased Fracture Risk in Women Treated With Aromatase Inhibitors Versus Tamoxifen: Beneficial Effect of Bisphosphonates. J Bone Miner Res. 2019.

19. Ramchand SK, Cheung YM, Yeo B, Grossmann M. The effects of adjuvant endocrine therapy on bone health in women with breast cancer. J Endocrinol. 2019;241(3):R111-R24.

20. Recine F, Bongiovanni A, Foca F, Mercatali L, Fausti V, Calpona S, et al. BoOne heealth manageement in patients with early breast cancer: A retrospective italian osteoncology center “Real-life” experience (BOHEME study). J Clin Med. 2019;8(11).

21. Stumpf U, Kostev K, Kyvernitakis J, Böcker W, Hadji P. Incidence of fractures in young women with breast cancer - a retrospective cohort study. J Bone Oncol. 2019;18.

22. Kim M, Kim H, Ahn SH, Tabatabaie V, Choi SW, Sohn G, et al. Changes in bone mineral density during 5 years of adjuvant treatment in premenopausal breast cancer patients. Breast Cancer Res Treat. 2020;180(3):657-63.

23. Lee J, Alqudaihi HM, Kang MS, Kim J, Lee JW, Ko BS, et al. Effect of Tamoxifen on the Risk of Osteoporosis and Osteoporotic Fracture in Younger Breast Cancer Survivors: A Nationwide Study. Front Oncol. 2020;10.

24. Reinhorn D, Yerushalmi R, Moore A, Desnoyers A, Saleh RR, Amir E, et al. Evolution in the risk of adverse events of adjuvant endocrine therapy in postmenopausal women with early-stage breast cancer. 2020.

25. Song Y, Xu YL, Lin Y, Zhao B, Sun Q. Fractures due to Aromatase Inhibitor Therapy for Breast Cancer: A Real-World Analysis of FAERS Data in the Past 15 Years. Oncol Res Treat. 2020;43(3):96-102.

26. Stumpf U, Kostev K, Siebenbürger G, Böcker W, Hadji P. Influence of chemotherapy and endocrine treatment on fractures in postmenopausal women with breast cancer – a retrospective cohort study. J Bone Oncol. 2020.

27. Yip CHW, Liem GS, Mo FKF, Pang E, Lei YY, Li L, et al. Bone Health in Premenopausal Chinese Patients after Adjuvant Chemotherapy for Early Breast Cancer. Breast Care. 2020.

28. Catalano A, Gaudio A, Morabito N, Basile G, Agostino RM, Xourafa A, et al. Quantitative ultrasound and DXA measurements in aromatase inhibitor-treated breast cancer women receiving denosumab. J Endocrinol Invest. 2017;40(8):851-7.

29. Catalano A, Morabito N, Agostino RM, Basile G, Gaudio A, Atteritano M, et al. Bone health assessment by quantitative ultrasound and dual-energy x-ray absorptiometry in postmenopausal women with breast cancer receiving aromatase inhibitors. Menopause. 2017;24(1):85-91.

30. Hans D, Šteňová E, Lamy O. The Trabecular Bone Score (TBS) Complements DXA and the FRAX as a Fracture Risk Assessment Tool in Routine Clinical Practice. Curr Osteoporosis Rep. 2017;15(6):521-31.

31. Hong AR, Kim JH, Lee KH, Kim TY, Im SA, Kim TY, et al. Long-term effect of aromatase inhibitors on bone microarchitecture and macroarchitecture in non-osteoporotic postmenopausal women with breast cancer. Osteoporosis Int. 2017;28(4):1413-22.

32. Mariotti V, Page DB, Davydov O, Hans D, Hudis CA, Patil S, et al. Assessing fracture risk in early stage breast cancer patients treated with aromatase-inhibitors: An enhanced screening approach incorporating trabecular bone score. J Bone Oncol. 2017;7:32-7.

33. Ramchand SK, Seeman E, Wang XF, Ghasem-Zadeh A, Francis PA, Ponnusamy EJ, et al. Premenopausal women with early breast cancer treated with estradiol suppression have severely deteriorated bone microstructure. Bone. 2017;103:131-5.

34. Rodriguez-Sanz M, Pineda-Moncusi M, Garcia-Giralt N, Servitja S, Martos T, Blanch-Rubio J, et al. TBS variation in breast cancer women completing AI-therapy: a prospective study of the B-able cohort. Journal of bone and mineral research Conference: 2016 annual meeting of the american society for bone and mineral research, ASBMR 2016 United states. 2017;31(Supplement 1) (no pagination).

35. Pineda-Moncusí M, Rodríguez-Sanz M, Servitja S, Díez-Pérez A, Tusquets I, Nogués X, et al. Study of the genetic basis of Trabecular Bone Score reduction related to aromatase inhibitors. Rev Osteoporosis Metab Miner. 2018;10(2):82-7.

36. Catalano A, Gaudio A, Agostino RM, Morabito N, Bellone F, Lasco A. Trabecular bone score and quantitative ultrasound measurements in the assessment of bone health in breast cancer survivors assuming aromatase inhibitors. J Endocrinol Invest. 2019.

37. Hopson MB, Onishi M, Awad D, Buono D, Maurer M, Crew KD, et al. Prospective Study Evaluating Changes in Bone Quality in Premenopausal Women With Breast Cancer Undergoing Adjuvant Chemotherapy. Clin Breast Cancer. 2020.

38. Schaffler-Schaden D, Kneidinger C, Schweighofer-Zwink G, Flamm M, Iglseder B, Pirich C. Evaluation of baseline fracture risk in younger postmenopausal women with breast cancer using different risk assessment methods. Skelet Radiol. 2020;49(6):1015-9.

39. Blok EJ, Roep JR, Meershoek-Lein Ranenbarg EM, Duym-de Carpentier M, Putter H, Van Den Bosch J, et al. Safety assessment of extended adjuvant endocrine therapy with letrozole; results of the randomized phase III IDEAL trial (BOOG 2006-05). Cancer research. 2017;77(4).

40. Mamounas EP, Bandos H, Lembersky BC, Geyer CE, Fehrenbacher L, Graham ML, et al. A randomized, doubleblinded, placebo-controlled clinical trial of extended adjuvant endocrine therapy (tx) with letrozole (L) in post-menopausal women with hormone-receptor (+) breast cancer (BC) who have completed previous adjuvant tx with an aromatase inhibitor (AI): results from NRG Oncology/NSABP B-42. Cancer research. 2017;77(4).

41. Blok EJ, Kroep JR, Kranenbarg EMK, Duijm-De Carpentier M, Putter H, Van Den Bosch J, et al. Optimal duration of extended adjuvant endocrine therapy for early breast cancer; results of the IDEAL trial (BOOG 2006-05). J Natl Cancer Inst. 2018;110(1):40-8.

42. Blok EJ, Kroep JR, Meershoek-Klein Kranenbarg E, Duijm-de Carpentier M, Putter H, Liefers GJ, et al. Treatment decisions and the impact of adverse events before and during extended endocrine therapy in postmenopausal early breast cancer. Eur J Cancer. 2018;95:59-67.

43. Colleoni M, Luo W, Karlsson P, Chirgwin J, Aebi S, Jerusalem G, et al. Extended adjuvant intermittent letrozole versus continuous letrozole in postmenopausal women with breast cancer (SOLE): a multicentre, open-label, randomised, phase 3 trial. Lancet Oncol. 2018;19(1):127-38.

44. Gnant M, Steger G, Greil R, Fitzal F, Mlineritsch B, Manfreda D, et al. A prospective randomized multi-center phase-III trial of additional 2 versus additional 5 years of anastrozole after initial 5 years of adjuvant endocrine therapy - Results from 3,484 postmenopausal women in the ABCSG-16 trial. Cancer research. 2018;78(4).

45. Goldvaser H, Barnes TA, Seruga B, Cescon DW, Ocana A, Ribnikar D, et al. Toxicity of Extended Adjuvant Therapy With Aromatase Inhibitors in Early Breast Cancer: A Systematic Review and Meta-analysis Review. 2018.

46. Van Hellemond I, Smorenburg CH, Peer P, Swinkels A, Seynaeve CM, Van Der Sangen M, et al. Assessment and management of bone health in women treated with adjuvant anastrozole in the DATA study. Journal of clinical oncology. 2018;36(15).

47. Qian X, Li Z, Ruan GD, Tu C, Ding W. Efficacy and toxicity of extended aromatase inhibitors after adjuvant aromatase inhibitors-containing therapy for hormone-receptor-positive breast cancer: a literature-based meta-analysis of randomized trials. Breast Cancer Res Treat. 2019.

48. van Hellemond IEG, Smorenburg CH, Peer PGM, Swinkels ACP, Seynaeve CM, van der Sangen MJC, et al. Assessment and management of bone health in women with early breast cancer receiving endocrine treatment in the DATA study. Int J Cancer. 2019;145(5):1325-33.

49. Xu L, Zhang Z, Xiang Q, Liu Q, Duan X, Liu Y, et al. Extended Adjuvant Therapy With Aromatase Inhibitors for Early Breast Cancer: A Meta-analysis of Randomized Controlled Trials. Clin Breast Cancer. 2019;19(5):e578-e88.

50. Mamounas EP, Bandos H, Lembersky BC, Jeong JH, Geyer CE, Rastogi P, et al. Ten-year results from NRG Oncology/NSABP B-42: a randomized, double-blinded, placebo-controlled clinical trial of extended adjuvant endocrine therapy with letrozole (L) in postmenopausal women with hormone-receptor+ breast cancer (BC) who have completed previous adjuvant therapy with an aromatase inhibitor (AI). Cancer research. 2020;80(4).

51. Pedersini R, Monteverdi S, Mazziotti G, Amoroso V, Roca E, Maffezzoni F, et al. Morphometric vertebral fractures in breast cancer patients treated with adjuvant aromatase inhibitor therapy: A cross-sectional study. Bone. 2017;97:147-52.

52. de Paulo TRS, Winters-Stone KM, Viezel J, Rossi FE, Simões RR, Tosello G, et al. Effects of resistance plus aerobic training on body composition and metabolic markers in older breast cancer survivors undergoing aromatase inhibitor therapy. Exp Gerontol. 2018;111:210-7.

53. Fong SSM, Choi AWM, Luk WS, Yam TTT, Leung JCY, Chung JWY. Bone Mineral Density, Balance Performance, Balance Self-Efficacy, and Falls in Breast Cancer Survivors With and Without Qigong Training: An Observational Study. Integr Cancer Ther. 2018;17(1):124-30.

54. Hsieh E, Wang Q, Zhang R, Niu X, Xia W, Fraenkel L, et al. Vertebral fractures among breast cancer survivors in China: A cross-sectional study of prevalence and health services gaps. BMC Cancer. 2018;18(1).

55. Prawiradilaga RS, Gunmalm V, Lund-Jacobsen T, Helge EW, Brøns C, Andersson M, et al. FRAX Calculated without BMD Resulting in a Higher Fracture Risk Than That Calculated with BMD in Women with Early Breast Cancer. J Osteoporosis. 2018;2018.

56. Dieckmeyer M, Ruschke S, Rohrmeier A, Syväri J, Einspieler I, Seifert-Klauss V, et al. Vertebral bone marrow fat fraction changes in postmenopausal women with breast cancer receiving combined aromatase inhibitor and bisphosphonate therapy. BMC Musculoskelet Disord. 2019;20(1).

57. Infante M, Fabi A, Cognetti F, Gorini S, Caprio M, Fabbri A. RANKL/RANK/OPG system beyond bone remodeling: Involvement in breast cancer and clinical perspectives. J Exp Clin Cancer Res. 2019;38(1).

58. Leslie WD, Morin SN, Lix LM, Niraula S, McCloskey EV, Johansson H, et al. Performance of FRAX in Women with Breast Cancer Initiating Aromatase Inhibitor Therapy: A Registry-Based Cohort Study. J Bone Miner Res. 2019.

59. Pedersini R, Amoroso V, Maffezzoni F, Gallo F, Turla A, Monteverdi S, et al. Association of Fat Body Mass with Vertebral Fractures in Postmenopausal Women with Early Breast Cancer Undergoing Adjuvant Aromatase Inhibitor Therapy. JAMA Netw Open. 2019;2(9).

60. García-Giralt N, Pineda-Moncusí M, Ovejero D, Aymar I, Soldado-Folgado J, Campodarve I, et al. Risk factors for incident fracture in patients with breast cancer treated with aromatase inhibitors: B-ABLE cohort. Rev Osteoporosis Metab Miner. 2020;12(1):7-13.

61. Yao S, Laurent CA, Roh JM, Lo J, Tang L, Hahn T, et al. Serum bone markers and risk of osteoporosis and fragility fractures in women who received endocrine therapy for breast cancer: a prospective study. Breast Cancer Res Treat. 2020;180(1):187-95.

62. Bošković L, Gašparić M, Petković M, Gugić D, Lovasić IB, Soldić Ž, et al. Bone health and adherence to vitamin D and calcium therapy in early breast cancer patients on endocrine therapy with aromatase inhibitors. Breast. 2017;31:16-9.

63. Sanmugarajah J, Allan S, Bagchi R, Laakso EL. Can a supervised exercise program compared to usual care prevent aromatase inhibitor-induced musculoskeletal pain in women with breast cancer? Cancer research. 2017;77(4).

64. Thomas GA, Cartmel B, Harrigan M, Fiellin M, Capozza S, Zhou Y, et al. The effect of exercise on body composition and bone mineral density in breast cancer survivors taking aromatase inhibitors. Obesity. 2017;25(2):346-51.

65. Baker MK, Peddle-McIntyre CJ, Galvão DA, Hunt C, Spry N, Newton RU. Whole Body Vibration Exposure on Markers of Bone Turnover, Body Composition, and Physical Functioning in Breast Cancer Patients Receiving Aromatase Inhibitor Therapy: A Randomized Controlled Trial. Integr Cancer Ther. 2018;17(3):968-78.

66. Peppone LJ, Ling M, Huston AJ, Reid ME, Janelsins MC, Puzas JE, et al. The effects of high-dose calcitriol and individualized exercise on bone metabolism in breast cancer survivors on hormonal therapy: a phase II feasibility trial. Supportive Care Cancer. 2018;26(8):2675-83.

67. Tanaka M, Itoh S, Takeuchi Y. Effectiveness of bisphosphonate combined with activated vitamin D in patients with aromatase inhibitor-induced osteoporosis after breast cancer operation. Osteoporos Sarcopenia. 2018;4(3):102-8.

68. Nct. Dietary and Exercise Interventions in Reducing Side Effects in Patients With Stage I-IIIa Breast Cancer Receiving Aromatase Inhibitors. <https://clinicaltrialsgov/show/NCT03953157>. 2019.

69. Nct. Effects of Progressive Relaxation Training in Breast Cancer Survivors Receiving Aromatatase Inhibitor Therapy. <https://clinicaltrialsgov/show/NCT04163692>. 2019.

70. Peppone LJ, Reschke JE, Janelsins MC, Inglis JE, Mustian KM, Culakova E, et al. A phase II RCT of high-dose vitamin D supplementation and exercise for cancer treatment-induced bone loss in breast cancer patients on aromatase inhibitors. Journal of clinical oncology. 2019;37.

71. Tabatabai LS, Bloom J, Stewart S, Sellmeyer DE. A Randomized Controlled Trial of Exercise to Prevent Bone Loss in Premenopausal Women with Breast Cancer. J Women's Health. 2019;28(1):87-92.

72. Saito T, Ono R, Kono S, Asano M, Fukuta A, Tanaka Y, et al. Physical activity among patients with breast cancer receiving aromatase inhibitors is associated with bone health: a cross-sectional observational study. Breast Cancer Res Treat. 2020.

73. Peppone L, Reschke J, Janelsins M, Inglis J, Mustian K, Culakova E, et al. High-dose vitamin d supplementation and exercise for cancer-treatment-induced bone loss in breast cancer patients on aromatase inhibitors: a phase II RCT. Supportive care in cancer. 2019;27(1):S45‐.

74. Gnant M, Van Poznak C, Schnipper L. Therapeutic Bone-Modifying Agents in the Nonmetastatic Breast Cancer Setting: The Controversy and a Value Assessment. Am Soc Clin Oncol Educ Book. 2017;37:116-22.

75. Lipton A, Chapman JA, Leitzel K, Garg A, Pritchard KI, Ingle JN, et al. Osteoporosis therapy and outcomes for postmenopausal patients with hormone receptor-positive breast cancer: NCIC CTG MA.27. Cancer. 2017;(no pagination).

76. Livi L, Saieva C, Desideri I, Scotti V, De Luca Cardillo C, Carta G, et al. A single-blind, randomized, placebo-controlled phase II study to evaluate the impact of oral ibandronate on bone mineral density in osteopenic breast cancer patients receiving adjuvant aromatase inhibitors: final results of the single-center BONADIUV trial. Cancer research. 2017;77(4).

77. Powles TJ, Paterson AHG, Gralow JR. Optimal use of adjuvant bisphosphonates and breast cancer. J Clin Oncol. 2017;35(23):2719-20.

78. Sestak I, Cuzick J, Blake G, Patel R, Coleman R, Eastell R. Effect of risedronate on bone loss due to anastrozole given to prevent breast cancer: 5-year results from the IBISII prevention trial. Journal of bone and mineral research. 2017;31.

79. Hirano A, Inoue H, Ogura K, Hattori A, Yukawa H, Sakaguchi S, et al. Long-term effect of exemestane therapy on bone mineral density supported by bisphosphonates: Results of 5-year adjuvant treatment in postmenopausal women with early-stage breast cancer. Asia-Pac J Clin Oncol. 2018;14(5):e238-e42.

80. Kyvernitakis I, Kann PH, Thomasius F, Hars O, Hadji P. Prevention of breast cancer treatment-induced bone loss in premenopausal women treated with zoledronic acid: Final 5-year results from the randomized, double-blind, placebo-controlled ProBONE II trial. Bone. 2018;114:109-15.

81. Pineda-Moncusí M, Servitja S, Casamayor G, Cos ML, Rial A, Rodriguez-Morera J, et al. Bone health evaluation one year after aromatase inhibitors completion. Bone. 2018;117:54-9.

82. Santa-Maria CA, Bardia A, Blackford AL, Snyder C, Connolly RM, Fetting JH, et al. A phase II study evaluating the efficacy of zoledronic acid in prevention of aromatase inhibitor-associated musculoskeletal symptoms: the ZAP trial. Breast Cancer Res Treat. 2018;171(1):121-9.

83. Wilson C, Bell R, Hinsley S, Marshall H, Brown J, Cameron D, et al. Adjuvant zoledronic acid reduces fractures in breast cancer patients; an AZURE (BIG 01/04) study. Eur J Cancer. 2018;94:70-8.

84. Kumari R, James E, Jose WM. Assessment of aromatase inhibitor-induced bone loss and appropriateness of supportive therapy in postmenopausal breast cancer patients at a tertiary care center. J Pharmacol Pharmather. 2019;10(4):118-25.

85. Sestak I, Blake GM, Patel R, Coleman RE, Cuzick J, Eastell R. Comparison of risedronate versus placebo in preventing anastrozole-induced bone loss in women at high risk of developing breast cancer with osteopenia. Bone. 2019;124:83-8.

86. Kong SH, Kim JH, Kim SW, Shin CS. Aromatase inhibitors attenuate the effect of alendronate in women with breast cancer. J Bone Miner Metab. 2020.

87. Nct. Efficacy of Denosumab on Normal BMD in Women Receiving Adjuvant Aromatase Inhibitors for Early Breast Cancer. <https://clinicaltrialsgov/show/NCT03324932>. 2017.

88. Nakamura Y, Kamimura M, Morikawa A, Taguchi A, Suzuki T, Kato H. Significant improvement of bone mineral density by denosumab treatment in japanese osteoporotic patients following breast cancer treatment. Ther Clin Risk Manage. 2018;14:543-9.

89. Nakatsukasa K, Koyama H, Ouchi Y, Sakaguchi K, Fujita Y, Matsuda T, et al. Effect of denosumab administration on low bone mineral density (T-score −1.0 to −2.5) in postmenopausal Japanese women receiving adjuvant aromatase inhibitors for non-metastatic breast cancer. J Bone Miner Metab. 2018;36(6):716-22.

90. Nakatsukasa K, Koyama H, Ouchi Y, Sakaguchi K, Fujita Y, Matsuda T, et al. Effect of denosumab on bone mineral density in Japanese women with osteopenia treated with aromatase inhibitors for breast cancer: Subgroup analyses of a Phase II study. Ther Clin Risk Manage. 2018;14:1213-8.

91. Galvano A, Scaturro D, Badalamenti G, Incorvaia L, Rizzo S, Castellana L, et al. Denosumab for bone health in prostate and breast cancer patients receiving endocrine therapy? A systematic review and a meta-analysis of randomized trials. J Bone Oncol. 2019;18.

92. Gnant M, Pfeiler G, Frantal S. Denosumab in early-stage breast cancer – Authors' reply. Lancet Oncol. 2019;20(5):236.

93. Gonzalez-Rodriguez E, Aubry-Rozier B, Stoll D, Zaman K, Lamy O. Sixty spontaneous vertebral fractures after denosumab discontinuation in 15 women with early-stage breast cancer under aromatase inhibitors. Breast Cancer Res Treat. 2019.

94. Lippman M. Adjuvant denosumab in postmenopausal patients with hormone receptor-positive breast cancer. Lancet Oncol. 2019;20(3):312-3.

95. Nakatsukasa K, Koyama H, Ouchi Y, Ono H, Sakaguchi K, Matsuda T, et al. Effect of denosumab on low bone mineral density in postmenopausal Japanese women receiving adjuvant aromatase inhibitors for non-metastatic breast cancer: 24-month results. Breast Cancer. 2019;26(1):106-12.

96. Nakatsukasa K, Koyama H, Ouchi Y, Sakaguchi K, Fujita Y, Matsuda T, et al. Effects of denosumab on bone mineral density in Japanese women with osteoporosis treated with aromatase inhibitors for breast cancer. J Bone Miner Metab. 2019;37(2):301-6.

97. Nakatsukasa K, Koyama H, Ouchi Y, Sakaguchi K, Fujita Y, Matsuda T, et al. Predictive factors for the efficacy of denosumab in postmenopausal Japanese women with non-metastatic breast cancer receiving adjuvant aromatase inhibitors: a combined analysis of two prospective clinical trials. J Bone Miner Metab. 2019;37(5):864-70.

98. Sakaguchi K, Ono H, Nakatsukasa K, Ishikawa T, Hasegawa Y, Takahashi M, et al. Efficacy of denosumab for restoring normal bone mineral density in women receiving adjuvant aromatase inhibitors for early breast cancer. Medicine. 2019;98(32).

99. Galvano A, Scaturro D, Bazan V, Letizia Mauro G, Russo A. Reply to: Denosumab for bone health in prostate and breast cancer patients receiving endocrine therapy? A systematic review and a meta-analysis of randomized trials (Galvano et al., J Bone Oncol 2019; 18:100252). J Bone Oncol. 2020.

100. Gonzalez-Rodriguez E, Aubry-Rozier B, Stoll D, Zaman K, Lamy O. Commentary to “Denosumab for bone health in prostate and breast cancer patients receiving endocrine therapy? A systematic review and a meta-analysis of randomized trials” (Galvano et al. J Bone Oncol 2019; 18:100252). J Bone Oncol. 2020.

101. Irelli A, Sirufo MM, Scipioni T, de Pietro F, Pancotti A, Ginaldi L, et al. Breast cancer patients receiving denosumab during adjuvant aromatase inhibitors treatment: Who are the “inadequate responders” patients to denosumab? J B U ON. 2020;25(2):648-54.

102. Jprn U. The efficacy of the use of denosumab in the prevention of aromatase inhibitor-induced bone loss in postmenopausal women with hormone receptor-positive breast cancer who are currently on or will iniate an aromatase inhibitor in the adjuvant setting. <http://wwwwhoint/trialsearch/Trial2aspx?TrialID=JPRN-UMIN000027425>. 2017.

103. Vliek SB, Meershoek-Klein Kranenbarg E, Van Rossum AGJ, Tanis BC, Putter H, Van Der Velden AWG, et al. The efficacy and safety of the addition of ibandronate to adjuvant hormonal therapy in post-menopausal women with hormone-receptor positive early breast cancer. First results of the TEAM IIB trial (BOOG 2006-04). Cancer research. 2017;77(4).

104. Bouvard B, Chatelais J, Soulié P, Hoppé E, Saulnier P, Capitain O, et al. Osteoporosis treatment and 10 years' oestrogen receptor+ breast cancer outcome in postmenopausal women treated with aromatase inhibitors. Eur J Cancer. 2018;101:87-94.

105. Coleman RE, Collinson M, Gregory W, Marshall H, Bell R, Dodwell D, et al. Benefits and risks of adjuvant treatment with zoledronic acid in stage II/III breast cancer. 10 years follow-up of the AZURE randomized clinical trial (BIG 01/04). J Bone Oncol. 2018;13:123-35.

106. Perrone F, De Laurentiis M, de Placido S, Orditura M, Cinieri S, Riccardi F, et al. The HOBOE-2 multicenter randomized phase III trial in premenopausal patients with hormone-receptor positive early breast cancer comparing triptorelin plus either tamoxifen or letrozole or letrozole + zoledronic acid. Ann Oncol. 2018;29:viii704.

107. Gralow JR, Barlow WE, Paterson AHG, Miao JL, Lew DL, Stopeck AT, et al. Phase III randomized trial of bisphosphonates as adjuvant therapy in breast cancer: S0307. J Natl Cancer Inst. 2019.

108. Livi L, Scotti V, Desideri I, Saieva C, Cecchini S, Francolini G, et al. Phase 2 placebo-controlled, single-blind trial to evaluate the impact of oral ibandronate on bone mineral density in osteopenic breast cancer patients receiving adjuvant aromatase inhibitors: 5-year results of the single-centre BONADIUV trial. Eur J Cancer. 2019;108:100-10.

109. Meattini I, Scotti V, Desideri I, Saieva C, Visani L, Salvestrini V, et al. Oral ibandronate for osteopenic breast cancer patients receiving adjuvant aromatase inhibitors: secondary 5-year survival outcomes analysis of the single-center phase 2 BONADIUV trial. Cancer research. 2019;79(4).

110. Perrone F, De Laurentiis M, De Placido S, Orditura M, Cinieri S, Riccardi F, et al. Adjuvant zoledronic acid and letrozole plus ovarian function suppression in premenopausal breast cancer: HOBOE phase 3 randomised trial. Eur J Cancer. 2019.

111. Van Hellemond IE, Smorenburg CH, Peer PG, Swinkels AC, Seynaeve CM, Van Der Sangen MJ, et al. No impact of osteoporosis or bisphosphonate use for osteoporosis on breast cancer outcome: a sub-study of the DATA trial. Cancer research. 2019;79(4).

112. van Hellemond IEG, Smorenburg CH, Peer PGM, Swinkels ACP, Seynaeve CM, van der Sangen MJC, et al. Breast cancer outcome in relation to bone mineral density and bisphosphonate use: a sub-study of the DATA trial. Breast Cancer Res Treat. 2020;180(3):675-85.

113. Gnant M, Pfeiler G, Steger GG, Egle D, Greil R, Fitzal F, et al. Adjuvant denosumab in postmenopausal patients with hormone receptor-positive breast cancer (ABCSG-18): disease-free survival results from a randomised, double-blind, placebo-controlled, phase 3 trial. Lancet Oncol. 2019;20(3):339-51.

114. Coleman R, Finkelstein DM, Barrios C, Martin M, Iwata H, Hegg R, et al. Adjuvant denosumab in early breast cancer (D-CARE): an international, multicentre, randomised, controlled, phase 3 trial. Lancet Oncol. 2020;21(1):60-72.

115. Suarez-Almazor ME, Herrera R, Lei X, Chavez-MacGregor M, Zhao H, Giordano SH. Survival in older women with early stage breast cancer receiving low-dose bisphosphonates or denosumab. Cancer. 2020.

116. Gunmalm V, Jørgensen NR, Abrahamsen B, Schwarz P. Evidence for the prevention of bone loss in elderly and old early non-metastatic breast cancer patients treated with aromatase inhibitors. Eur Geriatr Med. 2017;8(5-6):408-12.

117. Trémollieres FA, Ceausu I, Depypere H, Lambrinoudaki I, Mueck A, Pérez-López FR, et al. Osteoporosis management in patients with breast cancer: EMAS position statement. Maturitas. 2017;95:65-71.

118. Bharatuar A, Kar M, Khatri S, Goswami V, Sarin R, Dawood S, et al. Practical consensus recommendaton for adjuvant bone-modifying agents in breast cancer. South Asian J Cancer. 2018;7(2):91-5.

119. Fukumoto S, Matsumoto T. Cancer treatment-induced bone loss (CTIBL). Jpn J Cancer Chemother. 2018;45(12):1685-9.

120. Grossmann M, Ramchand SK, Milat F, Vincent A, Lim E, Kotowicz MA, et al. Assessment and management of bone health in women with oestrogen receptor-positive breast cancer receiving endocrine therapy: Position statement of the Endocrine Society of Australia, the Australian and New Zealand Bone & Mineral Society, the Australasian Menopause Society and the Clinical Oncology Society of Australia. Clin Endocrinol. 2018;89(3):280-96.

121. Handforth C, D’Oronzo S, Coleman R, Brown J. Cancer Treatment and Bone Health. Calcif Tissue Int. 2018;102(2):251-64.

122. Heeke A, Nunes MR, Lynce F. Bone-Modifying Agents in Early-Stage and Advanced Breast Cancer. Curr Breast Cancer Rep. 2018;10(4):241-50.

123. Jerzak KJ, Raphael J, Desautels DN, Blanchette PS, Tyono I, Pritchard KI. Bone-Targeted Therapy in Early Breast Cancer. Oncology (Williston Park, N Y ). 2018;32(11):562-9.

124. Lüftner D, Niepel D, Steger GG. Therapeutic approaches for protecting bone health in patients with breast cancer. Breast. 2018;37:28-35.

125. Rachner TD, Coleman R, Hadji P, Hofbauer LC. Bone health during endocrine therapy for cancer. Lancet Diabetes Endocrinol. 2018;6(11):901-10.

126. Ruzycki SM, Nixon NA. Bone health after diagnosis of breast cancer. CMAJ. 2018;190(49):E1452.

127. Sousa S, Clézardin P. Bone-Targeted Therapies in Cancer-Induced Bone Disease. Calcif Tissue Int. 2018;102(2):227-50.

128. Taguchi T. [Bone and calcium metabolism associated with malignancy. Clinical Characteristics and Treatment of Cancer Treatment Induced Bone Loss(CTIBL)in Breast Cancer.] Japanese. Clin calcium. 2018;28(11):1515-7.

129. Tseng OL, Spinelli JJ, Gotay CC, Ho WY, McBride ML, Dawes MG. Promoting bone health management in women diagnosed with breast cancer: a pilot randomized controlled trial. Arch Osteoporosis. 2018;13(1).

130. Biver E. Bone effects of bisphosphonates and denosumab treatments in breast or prostate cancer. Rev Med Suisse. 2019;15(647):824-30.

131. Bouvard B, Confavreux CB, Briot K, Bonneterre J, Cormier C, Cortet B, et al. French recommendations on strategies for preventing and treating osteoporosis induced by adjuvant breast cancer therapies. Jt Bone Spine. 2019;86(5):542-53.

132. Chukir T, Liu Y, Farooki A. Antiresorptive agents' bone-protective and adjuvant effects in postmenopausal women with early breast cancer. Br J Clin Pharmacol. 2019;85(6):1125-35.

133. Coleman R. Clinical benefits of bone targeted agents in early breast cancer. Breast. 2019;48:S92-S6.

134. Grossmann M, Ramchand SK, Milat F, Vincent A, Lim E, Kotowicz MA, et al. Assessment and management of bone health in women with oestrogen receptor-positive breast cancer receiving endocrine therapy: position statement summary. Med J Aust. 2019;211(5):224-9.

135. Gyori DJ, Bullington SM, Crawford BS, Vernon VP. Evaluation of appropriate use of bisphosphonates and denosumab in patients with cancer. J Oncol Pharm Pract. 2019.

136. Ottewell P, Wilson C. Bone-Targeted Agents in Breast Cancer: Do We Now Have All the Answers? Breast Cancer Basic Clin Res. 2019;13.

137. Paschou SΑ, Augoulea A, Lambrinoudaki I. Bone health care in women with breast cancer. Horm. 2019.

138. Ramchand SK, Cheung YM, Grossmann M. Bone health in women with breast cancer. Climacteric. 2019:1-7.

139. Razaq A, Khan S, Hassan J, Malik BH, Razaq M. Comparing the Efficacy and Safety of Denosumab with Bisphosphonates in Increasing Bone Mineral Density in Patients with Prostate Cancer and Breast Cancer on Antihormonal Treatment. Cureus. 2019;11(12):e6401.

140. Shapiro CL, Lacchetti C, Neuner J. Management of osteoporosis in survivors of adult cancers with nonmetastatic disease: ASCO clinical practice guideline summary. J Oncol Pract. 2019;15(11):665-9.

141. von Moos R, Costa L, Gonzalez-Suarez E, Terpos E, Niepel D, Body JJ. Management of bone health in solid tumours: From bisphosphonates to a monoclonal antibody. Cancer Treat Rev. 2019;76:57-67.

142. Fukumoto S, Soen S, Taguchi T, Ishikawa T, Matsushima H, Terauchi M, et al. Management manual for cancer treatment-induced bone loss (CTIBL): position statement of the JSBMR. J Bone Miner Metab. 2020;38(2):141-4.

143. Miyashita H, Satoi S, Kuno T, Cruz C, Malamud S, Kim SM. Bone modifying agents for bone loss in patients with aromatase inhibitor as adjuvant treatment for breast cancer; insights from a network meta-analysis. Breast Cancer Res Treat. 2020;181(2):279-89.

144. Neyro JL, Cristóbal I, Palacios S. Bone protection during breast cancer treatment. Rev Osteoporosis Metab Miner. 2020;12(1):3-6.
